# Phylogenetic informativeness reconciles ray-finned fish molecular divergence times

**DOI:** 10.1186/s12862-014-0169-0

**Published:** 2014-08-08

**Authors:** Alex Dornburg, Jeffrey P Townsend, Matt Friedman, Thomas J Near

**Affiliations:** 1 Department of Ecology and Evolutionary Biology, Yale University, New Haven 06520, Connecticut, USA; 2 Department of Biostatistics, Yale University, New Haven 06510, Connecticut, USA; 3 Program in Computational Biology and Bioinformatics, Yale University, New Haven 06510, Connecticut, USA; 4 Department of Earth Sciences, University of Oxford, South Parks Road, Oxford OX1 3AN, UK; 5 Yale Peabody Museum of Natural History, New Haven 06520, Connecticut, USA

**Keywords:** Molecular clock, Actinopterygii, Nucleotide Saturation, Homoplasy

## Abstract

**Background:**

Discordance among individual molecular age estimates, or between molecular age estimates and the fossil record, is observed in many clades across the Tree of Life. This discordance is attributed to a variety of variables including calibration age uncertainty, calibration placement, nucleotide substitution rate heterogeneity, or the specified molecular clock model. However, the impact of changes in phylogenetic informativeness of individual genes over time on phylogenetic inferences is rarely analyzed. Using nuclear and mitochondrial sequence data for ray-finned fishes (Actinopterygii) as an example, we extend the utility of phylogenetic informativeness profiles to predict the time intervals when nucleotide substitution saturation results in discordance among molecular ages estimated.

**Results:**

We demonstrate that even with identical calibration regimes and molecular clock methods, mitochondrial based molecular age estimates are systematically older than those estimated from nuclear sequences. This discordance is most severe for highly nested nodes corresponding to more recent (i.e., Jurassic-Recent) divergences. By removing data deemed saturated, we reconcile the competing age estimates and highlight that the older mtDNA based ages were driven by nucleotide saturation.

**Conclusions:**

Homoplasious site patterns in a DNA sequence alignment can systematically bias molecular divergence time estimates. Our study demonstrates that PI profiles can provide a non-arbitrary criterion for data exclusion to mitigate the influence of homoplasy on time calibrated branch length estimates. Analyses of actinopterygian molecular clocks demonstrate that scrutiny of the time scale on which sequence data is informative is a fundamental, but generally overlooked, step in molecular divergence time estimation.

## Background

Observations of discordance between paleontological and molecular age estimates, or between ages estimated from different molecular datasets, are fairly common and have been observed in angiosperms [1]-[4], mammals [5]-[10], ray-finned fishes [11]-[15], and various other lineages across the Tree of Life [16],[17]. Multiple factors are invoked to explain conflicting molecular age estimates, including modeling of paleontological calibrations [18]-[24], nucleotide substitution rate heterogeneity [25],[26], and the appropriateness of the molecular clock model used [27],[28]. However, the impact of the phylogenetic informativeness of gene sequences on molecular divergence time estimates has received considerably less attention.

Sequences whose sites have experienced large numbers of substitutions, either as a function of a rapid rate of molecular evolution or the passage of long periods of evolutionary time, will likely exhibit a high frequency of homoplasious character states. This homoplasy is problematic because a high frequency of convergent nucleotide states can bias branch length estimation in phylogenetic analyses, as the rate of hidden substitutions will often be under- or overestimated [29]-[32]. However, distinguishing between homoplasy and other sources of discordance is challenging in empirical datasets. This is largely because commonly used metrics that address the influence of nucleotide saturation on phylogenetic branch length estimates, such as saturation plots [33]-[35], are often difficult to interpret in terms of where in a given clade’s history the branch length estimates or inferences of phylogenetic tree topology may be compromised [36].

Phylogenetic informativeness (PI) methods offer a strategy to directly relate nucleotide saturation in data sets to molecular divergence time studies [36]. Using the ratio of a rate of evolution to the optimal rate of evolution for phylogenetic inference at a particular time, PI profiles quantify informativeness. Briefly, this approach takes an inferred vector of site-specific rates and generates a normalized, asymptotic likelihood density for a true synapomorphy occurring at a historical time T under a given model of character evolution. It should be noted that this is a predictive likelihood based entirely on the site-rates inferred, not an assessment of the validity of any empirical result. Analysis of the full site-rate distribution provides a profile of phylogenetic informativeness that spans the entire temporal span of the focal phylogeny, illuminating the scope of phylogenetic signal attributable to the data through evolutionary time [36]. Although phylogenetic informativeness profiles make no direct statement regarding the degree of saturation present in a dataset, the decline of the informativeness profile that follows the peak has been dubbed a “rain shadow of noise,” where the likely quantity of homoplasy influencing a node is comparable to the drop from the peak of the PI profile to its height aligned to the node [37]. This decline at depth from the peak of the PI profile should not only provide insights into where in the temporal span of the phylogeny to expect an increase in phylogenetic noise for topological inference, but also should provide a predictive tool for assessing the potential for data sets to provide strongly supported yet discordant age estimates.

Determining whether estimated ages are the result of estimation biases, or substantial gaps in the fossil record, is especially critical in reconstructing the history of vertebrate diversification. Many analyses of mitogenomic datasets sampled from lineages that span deep evolutionary time scales have resulted in age estimates that are far older than the expectations from the fossil record [14],[38]-[41] and the rapid rate of nucleotide substitution that characterizes the vertebrate mitochondrial genome has prompted multiple investigators to question whether molecular age estimates obtained using mitogenomic data are the result of such biases [30],[42].

The discordance between molecular age estimates is striking within ray-finned fishes (Actinopterygii). While actinopterygians have a fossil record dating to the Devonian [43], limited paleobiological surveys of richness through time [44] and striking disagreement between competing molecular age estimates have impeded our understanding of the timescale underlying their diversification. Estimates made for actinopterygians on the basis of mitogenomic and nuclear datasets often indicate very different evolutionary timelines. For example, the origin of acanthomorphs (spiny-rayed fishes), which include nearly one third of all living vertebrates [45], is placed in the Triassic (252.2-201.3 Ma) based on mitogenomic age estimates [38],[40]. This suggests that the majority of living fish diversity originated during a period of recovery following the Permian-Triassic mass extinction event (252.2 Ma), with major acanthomorph lineages beginning to radiate throughout the Triassic and Jurassic (201.3-145.0 Ma). By contrast, recent molecular clock analyses using nDNA place the origin of acanthomorphs in the Early Cretaceous, followed by extensive diversification in the Late Cretaceous and early Paleocene [13],[46]-[49]. A comparable timescale has also been estimated by some mitogenomic analyses [50], and is more consistent with patterns in the fossil record [44],[51]-[53] than the early Mesozoic acanthomorph radiation implied by other studies. As actinopterygians comprise half of all living backboned animals, including several model organisms and species of great economic importance, understanding the timing of their diversification provides critical insight into the evolutionary history of vertebrates.

Disagreement between mtDNA and nDNA estimates is not directionally consistent, i.e., many mtDNA estimates are older than nDNA counterparts, but there are examples of the opposite pattern [13],[48],[50], and discordant timescales might also arise from other factors that differ between analyses: the selection and placement of calibrations, the clock models applied, and the taxa sampled. However, this study seeks to fix, as completely as possible, these other variables, and explore the impact that different sequence data have on estimation of evolutionary timescales. We evaluate phylogenetic informativeness (PI) profiles [36] for gene sequences sampled among major actinopterygian lineages and demonstrate that is not necessary to attribute divergent molecular age estimates to issues in modeling of paleontological data as calibration age priors as previsouly suggested [13]. Instead, the divergent estimates can be attributed to levels of homoplasy in the mtDNA and nuclear gene datasets that distort inferences at different time scales. By removing data partitions that are saturated, we reconcile divergent molecular age estimates for actinopterygians, bringing these more in line with ages implied by the fossil record. These results demonstrate that selection of sequence data appropriate for the time scale of inferences is as important as the selection of calibrations and molecular clock models for divergence time estimation.

## Results

**Figure 1:**
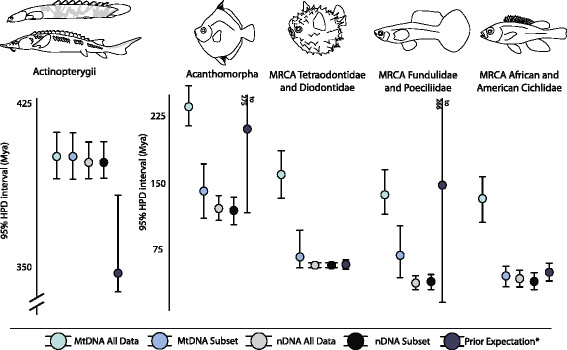
Comparison of the estimated 95% HPD interval for five example contrasts of mtDNA and nDNA age estimates from all data, those from datasets pruned of the saturated partitions, and the prior age expectation based on the effective prior (*analysis run without sequence data): Acanthomorpha (spiny-rayed fishes); MRCA Fundulidae and Poeciliidae (topminnows and livebearers); MRCA African and American cichlids, MRCA Tetraodontidae and Diodontidae (smooth and spiny puffers), and Actinopterygii (ray-finned fishes). Lines indicate bounds of the 95% HPD interval, circles correspond to mean age estimates.

**Figure 2:**
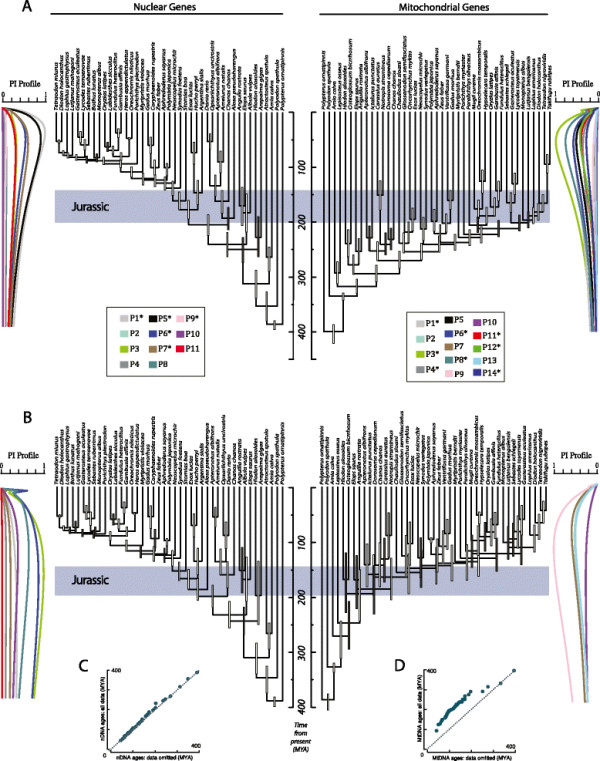
**Comparison of mtDNA and nDNA chronograms for actinopterygians.** Ages were estimated by analyzing **A)** all data and **B)** analyzing data that excluded saturated partitions. Bars indicate 95% HPD intervals of age estimates. Light bars indicate posterior probabilities greater than 0.95. Gray bars indicate posterior probabilities below 0.95. Phylogenetic informativeness profiles for both datasets are shown adjacent to the associated chronograms. Colors identify individual partitions. **C)** Comparison of mtDNA mean age estimates for all nodes when all data is used and the mean ages from the pruned dataset. **D)** Comparison of nDNA mean age estimates for all nodes when all data is used and the mean ages from the pruned dataset.

Information-theoretic based searches of partitioning strategies found 11 and 14 partitions as the best fit for the mtDNA (Table 1) and nuclear DNA (nDNA; Table 2) datasets respectively. Molecular age estimates between sets of analyses with differing calibration schemes were very similar (Figures 1 and 2; Additional file 1: Table S1 and S2) and substantially outside of the prior expectations (Figure 1). As manipulation of the calibration age priors to reflect potential uncertainties in the fossil record had a minimal influence on trends in the resulting molecular age estimates, we restrict discussion to the most calibration-rich analysis here. Regardless of calibration strategy, molecular dating analyses of the mtDNA and nDNA datasets including all data partitions resulted in very different posterior age estimates, with the mtDNA posterior age estimates generally being much older and exclusive of the 95% highest posterior density interval (HPD) for many nDNA estimates (Figures 1 and 2). Estimated ages were most consistent towards the root of the tree, with higher uncertainty in the mtDNA estimates. The HPD of the estimated age from the mtDNA analysis for the most recent common ancestor (MRCA) of actinopterygians ranged between 383 and 416 Ma (mean: 395 Ma [Middle Devonian]) similar to the HPD of ages for this node in the nDNA analysis, which ranged between 383 and 399 Ma (mean: 389 Ma [Middle Devonian]; Figure 1). In contrast, instances of discordance between the mtDNA and nDNA age estimates were more extreme within acanthomorphs. For example, the HPD for the MRCA of Tetraodontidae and Diodontidae (pufferfishes) ranged between 50 and 57 Ma (mean: 52 Ma [Eocene]) in the nDNA analyses, whereas the HPD ranged between 149 to 179 Ma (mean: 164 Ma [Middle Jurassic]) in the mtDNA analyses (Figure 1). Similarly, the HPD for the MRCA of *Fundulus* and *Gambusia* ranged between 124 and 160 Ma (mean: 141 Ma [Early Cretaceous]) in the mtDNA analyses, compared to an HPD between 29 and 46 Ma in the nDNA analyses (mean: 37 Ma [Eocene]; Figure 1).

**Table 1:** Best-fit nucleotide substitution models and partition strategies identified by PartitionFinder and peaks of phylogenetic informativeness for mtDNA

**Subset**	**Best model**	**Subset partitions**	**Subset sites**	**PI peak/slope**
**1**	**GTR + I + G**	***nd5_1, cytb_1, nd2_1, atp8_3***	**1-1145\3, 1832-2008\3, 6610-7665\3, 7668-9522\3**	**91/-3.61E-03**
2	GTR + I + G	*cytb_2, coi_2*	2-1145\3, 2010-3562\3	116/-2.60E-04
**3**	**GTR + I + G**	***cytb_3,nd5_3***	**3-1145\3, 7667-9522\3**	**72/-9.25E-03**
**4**	**GTR + I + G**	***nd5_2,atp8_1,nd2_2,atp6_1***	**1146-1831\3, 1833-2008\3, 6611-7665\3, 7666-9522\3**	**87/-2.36E-03**
**5**	**GTR + I + G**	***coiii_2,atp6_2,atp8_2***	**1147-1831\3, 1834-2008\3, 4255-5637\3**	**72/-4.74E-03**
**6**	**GTR + I + G**	***coiii_3,coiii_1,atp6_3***	**1148-1831\3, 4254-5637\3, 4256-5637\3**	**77/-4.85E-03**
7	SYM + I + G	*coi_1*	2009-3562\3	106/-3.56E-04
**8**	**GTR + G**	***coii_3,coi_3***	**2011-3562\3, 3565-4253\3**	**72/-7.35E-03**
9	SYM + I + G	*nd1_1,coii_1*	3563-4253\3, 5638-6609\3	145/-3.25E-04
10	GTR + I + G	*nd1_2,coii_2*	3564-4253\3, 5639-6609\3	217/-4.80E-05
**11**	**HKY + I + G**	***nd1_3***	**5640-6609\3**	**72/-4.29E-03**
**12**	**HKY + I + G**	***nd2_3***	**6612-7665\3**	**62/-4.75E-03**
13	GTR + I + G	*nd6_2*	9523-10061\3	116/-2.23E-04
**14**	**GTR + I + G**	***nd6_1,nd6_3***	**9524-10061\3, 9525-10061\3**	**72/-2.56E-03**

Values following underscores after the gene name indicate first, second, or third codon positions. Subset sites correspond to the order of genes in the concatenated alignment. Peak of the PI profiles are indicated in millions of years. Slope values correspond to the slope of a chord joining the peak of profile and the midpoint of the profile between the peak and the root of the tree. Bolded partitions indicate partitions removed for subsequent analysis.

**Table 2:** Best-fit nucleotide substitution models and partition strategies identified by PartitionFinder and peaks of phylogenetic informativeness for nDNA

**Subset**	**Best model**	**Subset partitions**	**Subset sites**	**PI peak/slope**
1	GTR + I + G	*Plag_2, SH3PX3_2, SREB2_2, ZIC1_1*	1-691\3, 2785-3497\3, 3500-4636\3, 5323-6194\3	180/-1.24e-04
**2**	**SYM + G**	***Plag_3, ptr_1, SREB2_3***	**2-691\3, 692-1405\3, 3498-4636\3**	**62/-7.79e-03**
3	HKY + I + G	*Plag_1, TBR1_3, TBR1_1, glyt_1*	3-691\3, 4638-5321\3, 4639-5321\3, 6197-7024\3	85/-1.29e-03
4	GTR + I + G	*ptr_2, myh6_2*	693-1405\3, 7027-7769\3	128/-3.82e-05
**5**	**GTR + I + G**	***ptr_3, glyt_3, myh6_3***	**694-1405\3, 6196-7024\3, 7025-7769\3**	**70/-4.70e-03**
6	SYM + I + G	*RAG1_2, RAG1_1*	1406-2784\3, 1408-2784\3	70/-1.46e-03
7	GTR + I + G	*RAG1_3, TBR1_2*	1407-2784\3, 4637-5321\3	245/-3.34e-05
**8**	**GTR + I + G**	***SH3PX3_3, myh6_1***	**2786-3497\3, 7026-7769\3**	**62/-1.93e-03**
**9**	**K80 + I + G**	***SH3PX3_1, SREB2_1***	**2787-3497\3, 3499-4636\3**	**58/-3.40e-03**
10	K80 + G	*ZIC1_3*	5322-6194\3	66/-4.55e-04
11	K80 + I + G	*ZIC1_2, glyt_2*	5324-6194\3, 6195-7024\3	163/-8.90e-06

Values following underscores after the gene name indicate first, second, or third codon positions. Subset sites correspond to the order of genes in the concatenated alignment. Peak of the PI profiles are indicated in millions of years. Slope values correspond to the slope of a chord joining the peak of profile and the midpoint of the profile between the peak and the root of the tree. Bolded partitions indicate partitions removed for subsequent analysis.

**Figure 3:**
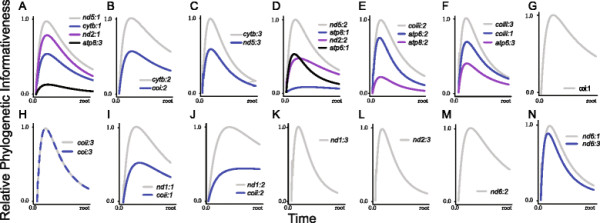
**Visualizations of the individual phylogenetic informativeness profiles for each codon position in the mtDNA dataset.** Inset letters **(A-N)** correspond to data partitions (1–14) in Table 1.

**Figure 4:**
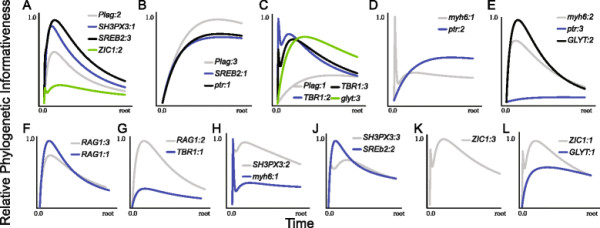
**Visualizations of the individual phylogenetic informativeness profiles for each relative codon position in the nDNA dataset.** The first frame of each relative codon position corresponds to starting position of each gene in the Near et al. [48] alignment. Inset letters **(A-L)** correspond to data partitions (1–11) in Table 2.

**Figure 5:**
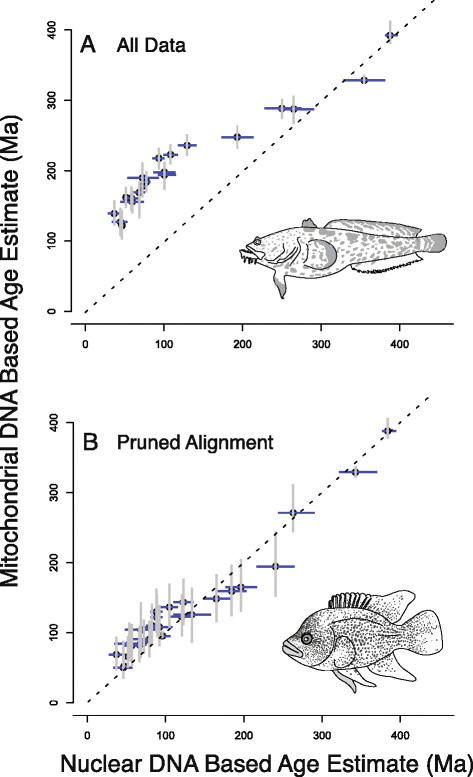
**Comparison of means and 95% HPD intervals for mtDNA and nDNA based posterior age estimates, (A) when all data from each dataset is included, and (B) when partitions are excluded from each dataset based on their PI profile.** Black circles indicate mean age estimates, gray and blue bars respectively indicate the 95% HPD interval of the mtDNA and nDNA based analyses.

The phylogenetic informativeness profile of several mtDNA partitions peaked prior to the majority of nodes present in the tree (Figure 2A; Table 1), and the shapes of the PI profiles for each individual codon position were similar within partitions (Figure 3). Higher profiles of PI indicate greater utility for phylogenetic inference. However, a decline of PI profile following the peak is indicative of a “rainshadow of noise”, reflecting an increased probability that numerous hidden substitutions have accumulated that can mislead phylogenetic inference [37]. While the informativeness profiles of the nuclear gene partitions also exhibited a signature of homoplasy (Table 2) and conservation of PI profile shape within partitions (Figure 4), these declines were much less severe than those observed for the mtDNA partitions (Figure 2A). Removal of nucleotide data partitions with PI profiles that exhibit greater than a five percent decay of informativeness from the PI profile peak prior to the Cretaceous-Jurassic boundary (145.0 Ma) from the subsequent relaxed molecular clock analyses removed 7354 individual sites (Table 1) and resulted in substantial changes to the posterior age estimates using mtDNA (Figures 2B and 5). Indeed, almost all estimated ages from the mtDNA dataset after pruning of saturated data partitions shifted between 50 and 100 million years towards the present (Figures 2C and 5B). In contrast, removal of the saturated nDNA data partitions resulted in a removal of 2710 individual sites (Table 2) that had less effect on divergence time estimates (Figures 2D and 5). This global shift in node age estimates undermines support for a more ancient timescale of ray-finned fish evolution, instead reconciling the divergence time estimates generated by the two datasets. While analysis of the complete mtDNA dataset estimated the bulk of extant ray-finned fish lineages to have originated in the Jurassic (Figures 2A and 5), analysis of datasets pruned of saturated partitions shifted the majority of these estimates into the Cretaceous (145.0-66.0 Ma), and Paleogene (Figures 2B and 5). For example the HPD estimated from the pruned mtDNA analysis for the MRCA of acanthomorphs shifted to a range between 112 and 179 Ma (mean: 145 Ma [Early Cretaceous]). This result is congruent with the HPD of 108–135 Ma based on nDNA (mean: 122 Ma; Figure 2B), but substantially deviates from the HPD of 223–254 Ma estimated using the full mtDNA dataset (mean: 238 Ma [Late Triassic]; Figure 2A). This shift in estimated ages was also observed in younger clades. For instance, the HPD of 34–89 Ma (mean: 50 Ma [Eocene]), estimated from the pruned mtDNA analysis for the MRCA of sticklebacks and eelpouts undermines the credibility for the original estimated HPD of 109–149 Ma (mean: 129 Ma [Early Cretaceous]) obtained from the full mtDNA dataset. Instead this result now largely overlaps with the nDNA-based age HPD of 33–58 Ma (Mean: 45 Ma [Eocene-Paleocene]; Figure 2B).

## Discussion

### The timescale of ray-finned fish evolution

Discordance between the older mtDNA age estimates and the more recent nDNA based estimates have been attributed to a lack of shared calibrations between mtDNA and nDNA based studies [13]. However, despite using identical calibrations between datasets, our mtDNA based estimates still support an origin of most major lineages in the Jurassic (Figure 2A). These results are similar to some previous mtDNA based studies [38], and are in direct opposition with patterns of fossil richness that depict a radiation of living actinopterygian, particularly species-rich acanthomorph, lineages in the Cretaceous [44],[52],[54],[55]. These ages are also at odds with our nDNA based age estimates, which more closely match paleontological expectations within acanthomorphs (Figure 2A). We therefore find little support that calibration choice is the primary driver of discordant age estimates between these datasets.

Instead, PI profiles show that for deep divergences more than half of the identified mtDNA protein-coding gene partitions predict potentially misleading saturation (Figures 2A and 3). Saturation of mitogenomic data has been suggested to bias topological inferences in ray-finned fishes [56]. Removal of partitions exhibiting predicted saturation resulted in a topological rearrangement that greatly diminished support for relationships that differed from the nDNA based topology globally. Even when taking uncertainty in phylogenetic relationships into account, removal of saturated partitions resulted in a consistent shift in age estimates by as much as 100 My towards the present for almost all nodes in the tree (Figure 1), with 95% HPD intervals overlapping between nDNA and mtDNA based estimates (Figure 5). In contrast, removal of noisy partitions from the nDNA analysis had a neglible affect on the resulting divergence times, as would be predicted by the shallow decline in the PI profiles (Table 2; Figure 4).

This reconciliation of the ray-finned fish evolutionary timeline contributes to a strengthened historical framework that promises new insights into the evolutionary processes that generate and maintain aquatic biodiversity. Our finding suggests that at least some of the discordance surrounding actinopterygian divergence time estimates [12]-[14],[38],[40],[57],[58] can be explained by branch length estimation biases. However, this finding should not be viewed as a problem restricted to mtDNA or as a general phenomenon in which saturation always results in older age estimates. The latter is certainly not true, as Phillips [59] demonstrated that depending on the calibration placement and the character state patterns of saturated sites, nucleotide saturation can result in either tree extension or compression.

Deep time mitogenomic studies of fishes also do not always conflict with nDNA based studies. For example, in a mitogenomic study by Miya et al. [50], a large range of sampled acanthomorph lineages have divergence time estimates that overlap between nDNA based [46]-[49] and reject previous mtDNA based analyses [40]. This reversal of conflict is partially explained for two reasons. First, our informativeness profiles show partitions of the fish mitochondrial genome to be more informative at recent timescales (Figure 3), and Miya et al. [50] limited their taxon sampling to only 30% of the timescale sampled by Miya et al. [40]. Second, this study excluded the gene nd6, from which we also exclude two thirds of codon positions in this study (Table 2), and recoded transitional changes (changes within purines or pyrimidines) to a single state in an effort to exclude saturated sites [50]. Although Miya et al. [40] also attempted to mitigate the influence of saturation by excluding third codon positions, our results highlight rates of molecular evolution do not always conform to codon positions and that informativeness for some first and second positions also declines over deep timescales (Figure 3).

Our reconciliation of the timeline of ray-finned fish diversification adds support for the growing consensus that the patterns of species richness observed in living fishes are largely the product of diversification during the late Mesozoic and Cenozoic [13],[47],[48],[53],[58],[60]. Much of the diversity of living fishes can be attributed to the success of acanthomorphs, which comprise roughly one in every three vertebrate species. Understanding the timeline underlying this group is therefore not only critical to investigations of the evolution of fish biodiversity, but also to investigations of vertebrate evolution in general. Although there is still conflict between divergence times estimated for acanthomorph clades such as tetraodontiforms [11],[15],[48],[49],[61]-[63], cichlids [47],[48],[64],[65], and notothenioids [48],[66],[67], uncertainties surrounding competing mean age estimates typically show broad overlap between the majority of studies. This is encouraging and suggests convergence on a robust temporal framework from which to unlock the mode and tempo of diversification in this spectacular group of vertebrates.

### Profiling phylogenetic informativeness

Recognition that convergence in nucleotide character states, or saturation, diminishes the utility of gene sequences for phylogenetic inference at deeper evolutionary time scales is not new [29],[31],[33],[35],[68]-[70], and multiple approaches exist to assess when homoplasy has or will critically influence phylogenetic inferences. These include saturation plots [31],[33], RY coding variable characters [71],[72], down weighting characters [72],[73], and site removal [70],[74]. However, the development of a predictive framework that facilitates careful scrutiny of the power of diverse datasets to resolve phylogenetic problems has only recently begun [36],[75]-[77]. Our finding that PI profiles predict the temporal optimality of markers for providing branch length estimates identifies a useful heuristic framework for assessing the credibility of existing age estimates.

While discordance in molecular age estimates is frequently attributed to factors such as differential application of fossil-based age constraints [78]-[81], suitable modeling of paleontological calibration data [18]-[20], nucleotide substitution rate heterogeneity [25],[26],[82], or differences between molecular clock models [27],[83]-[85], our results demonstrate that the choice of molecular markers can also drive discordance when identical analysis conditions are employed. Although our study focuses on largely on mitochondrial genes because these have been found to have a nucleotide substitution rate much higher than nuclear exons commonly used in phylogenetic studies of many vertebrate clades [86]-[88], this is not a universal pattern across the tree of life [89]-[92]. Nucleotide saturation is a feature often observed in next-generation phylogenomic datasets [73],[93]-[96].

By utilizing phylogenetic informativeness approaches to identify data partitions characterized by saturation and homoplasy, much of the discordance between mtDNA and nDNA datasets is reconciled (Figure 2). It should be noted that this reconciliation does not always guarantee a one-to-one mapping of divergence time estimates between analyses nor does it predict how changes in the analytical conditions will influence posterior age estimates. For example, our exclusion of a prior age calibration for the MRCA of African and New World cichlids or stem Tetraodontiformes resulted in a shift to slightly older ages when using the pruned mtDNA dataset while not having a pronounced affect on the distribution of the global node ages inferred in nDNA based analyses (Additional file 2: Figure S1 and Additional file 3: Figure S2). However, in this case differences between age estimates were minor, with the removal of saturated data partitions having a far greater impact on resulting age estimates.

Phylogenetic informativeness approaches facilitate a diagnosis of when to expect a rise in homoplasious site patterns, offering an objective criterion for screening data by its utility for molecular divergence time estimation. As phylogenetic datasets become “phylogenomic” in scale [97], development of these approaches becomes essential for the selection of loci that will not be misleading from potentially hundreds of markers [98]. Our results demonstrate that for age-estimation just as for phylogenetic inference [99], more data alone is not enough. While adding more data can provide novel insights into the evolutionary patterns that underlie the Tree of Life [100], more data alone does not render an analysis immune from methodological artifacts such as homoplasy [73],[94],[99],[101]-[103]. As divergence time estimates rely on calibrated substitution rate estimates our results underscore that it is necessary to diagnose the effects of saturation and homoplasy at different time scales, even as hundreds, if not thousands, of loci become applied to dating the Genomic Tree of Life.

## Conclusions

We demonstrate that even with identical calibration regimes and molecular clock methods, the influence of homoplasy has a pronounced affect on divergence time estimates. We also demonstrate the utility of PI profiles for providing a much-needed non-arbitrary criterion for data exclusion. By extending the utility of PI profiles to this task, we highlight the ability of these methods to assess the robustness of age estimates relative to the frequency of homoplasious character states in the data. Applying this approach to the timescale of ray-finned fish evolution, we reconcile two fundamentally different views on the timescale of aquatic vertebrate diversification by removing partitions deemed saturated. The results obtained by pruning the saturated positions in this manner are more in line with paleontological expectations, suggesting that most major lineages of extant fishes today are Cretaceous in origin rather than emerging subsequent to the Permian-Triassic mass extinction event or the remnants of an ancient Jurassic radiation. Our results suggest that in addition to care in the selection of calibrations and molecular clock models, careful scrutiny of the potentially misleading impact of homoplasious data to be a fundamental component of divergence time estimation.

## Methods

### Dataset assembly

All of the DNA sequence data used in this study was obtained from Genbank. The ray-finned fish nuclear gene dataset contained 9 nuclear protein-coding genes (*zic1, myh6, rag1, ptr, tbr1, Glyt, SH3PX3, plag12, sreb2*), sampled from 44 species that included representatives from most of the major ray-finned fish lineages (Additional file 1: Table S1). We contrasted inferences based on the nuclear gene dataset with alignments from 10 protein-coding mtDNA genes (*nd1*, *nd2*, *COI*, *CO2*, *atp8*, *atp6*, *COIII*, *nd5*, *nd6*, and *cytb*) for representatives of the same major ray-finned fish lineages (Additional file 1: Table S2). Alignments for each mtDNA and nuclear gene were generated using MUSCLE v3.7 [104], then refined by eye using the translated amino acid sequences. Individual gene alignment files were concatenated using Phyutility [105]. For both datasets, we simultaneously assessed optimal partitioning strategies and the fit of potential nucleotide substitution models by comparing Bayesian Information Criterion (BIC) scores calculated using PartitionFinder [106]. Potential partitioning strategies that were compared included all possible gene and codon partition schemes, ranging from a single global partition to allowing each gene and codon position to have their own partition.

### Divergence time estimation

We used BEAST v.1.7.5 [107] to infer the marginal posterior distribution of ultrametric trees under a model of uncorrelated rates that follow a lognormal distribution (UCLN) for all analyses. For each BEAST run, we assigned a birth-death prior to rates of cladogenesis [107] and ran four independent Markov Chain Monte Carlo (MCMC) runs between 100 million and 1 billion generations, sampling every 1000–10000 generations. Chains were deemed convergent by visual examination of the chain likelihoods (Additional file 4: Figure S3) in Tracer 1.5 [108]. To ensure adequate mixing of each chain, the effective sample sizes (ESS) for all model parameters were assessed with ESS values above 200 indicating appropriate sampling from the posterior distribution of each parameter.

We enforced the monophyly of several nodes in both sets of analyses, as this constraint greatly decreased the time to convergence in preliminary analysis. The monophyly of Actinopteri relative to *Polypterus* was enforced based on results of previously published sets of phylogenetic analyses of morphological and molecular data [43],[109]-[111]. Additionally the monophyly of teleosts, euteleosts, neopterygians, otocephalans, acanthopterygians, cyprinodontiforms, tetraodontiforms, cichlids, and acanthomorphs was enforced based on previous phylogenetic analyses [13],[46]-[49],[112],[113].

Divergence time estimates were calibrated in a series of three sets of analyses that used between five and seven calibrations previously utilized in investigations of actinopterygian divergence times [13],[48]-[50],[64]. Applying identical calibrations and analytical conditions to both datasets allowed us to directly investigate the potential for saturation to influence divergence time estimates. Multiple studies have demonstrated that the width of the calibration age prior distribution influences the posterior distribution of Bayesian age estimates [18],[19],[114], and upper bounds on priors were identical to those in Near et al. [13],[48] and Friedman et al. [64] for direct comparison. All analyses were run with and without nucleotide data to assess the influence of the prior on the posterior distribution of age estimates [27].

### Paleontological data

Seven potential calibrations based on paleontogical data were taken from Near et al. [13], Near et al. [48], and Friedman et al. [64]. (1) The most recent common ancestor (MRCA) of all crown Actinopterygii was calibrated based on the occurrence of †*Mimipiscis toombsi* and †*Moythomasia durgaringa* from the Gogo Formation of Western Australia [111], 382.5 Ma [115]. The 95% prior age interval was set to 419 Ma based on the appearance of †*Guiyu oneiros* which represents the minimal age for the MRCA of Actinopterygii and Sarcopterygii [116]. (2) Crown-group Actinopteri was calibrated based on the Mississippian taxon, †*Cosmoptychius striatus* from the Wardie Shales, Lower Oil Shale Group, Scotland [117]. We assigned an absolute age estimate of 325.5 Ma [13],[118] with 95% of the prior interval set to 373 Ma with a soft upper bound, based on the maximum age bracketing approach of Marshall [20]. Although some analyses place †*Cosmoptychius* within the actinopteran crown [43],[119], others suggest it is a stem actinopteran [111],[120] . The minimum age estimate used by Near et al. [13] and applied here for †*Cosmoptychius* corresponds to the mid-Serpukhovian of the revised geological timescale [121]. The Serpukhovian †*Discoserra* is widely recognized as a crown actinopteran [12],[120] , so the age estimate applied here is appropriate regardless of specific placement of †*Cosmoptychius*. (3) The MRCA of Holostei (*Amia* and *Atractosteus + Lepisosteus* in this study) was calibrated based on the appearance of †*Watsonulus eugnathoides* from the Middle Sakamena Formation of Madagascar [122] with a minimal age of 245.9 Ma [123],[124] and 95% soft upper bound of 311 Ma based on the age of †*Mesopoma planti*[13],[125]. (4) The stem polymixiiform †*Homonotichthys dorsalis* from the Cenomanian Lower Chalk of Sussex and Kent, United Kingdom [126] was used to calibrate the MRCA of Polymixiiformes and Percopsiformes with a minimum age of 93.6 Ma and a 95% prior density interval that spanned 99.6 Ma based on the appearance of the putative stem acanthomorphs †*Aulolepis*, †*Ctenothrissa* and †*Heterothrissa*[13],[126],[127]. (5) The MRCA of extant spiny and smooth pufferfishes (Diodontidae and Tetraodontidae) was calibrated based on the appearance of several stem diodontids including †*Prodiodon tenuispinus*, †*P. erinaceus*, †*Heptadiodon echinus*, and †*Zignodon fornasieroae* from Bolca, Italy [128]. We do not accept the diodontid dentition described by Gallo et al. [129] as a reliable fossil calibration for the divergence between Diodontidae and Tetraodontidae. A Maastrichtian age is proposed for the fossil based on its color and general locality, but there is no record of the geological horizon from which it was collected and no matrix remains adhered to the specimen that might better constrain provenance. Following Near et al. [13] we set the minimum age of this calibration to 50 Ma with 95% of the prior age interval set to 57.3 Ma based on the maximum age bracketting approach of Marshall [20]. For all the above, we utilized lognormal prior age intervals with soft upper bounds allowing ages to be sampled outside the prior distribution of age estimates [130]. We adopted two calibration strategies for the divergence between Tetraodontiformes and Lophiiformes (6). Plectocretacicoidea contains a set of morphologically diverse Late Cretaceous acanthomorphs that have been interpreted as stem tetraodontiforms [131],[132]. The oldest plectocretacicoid, the early Cenomanian *Plectocretacicus*, has been nominated as a key fossil calibration for animal phylogenies [23],[24]. However, the interpretation of anatomically similar, coeval armoured acanthomorphs from Mexico as beryciforms [133] raises questions about the affinities of *Plectocretacicus* specifically and plectocretacicoids generally. The youngest plectocretacicoid is *Cretatriacanthus*, which is best known from the latest Campanian-earliest Maastrichtian of Nardò, Italy. Based on the argumentation given by Friedman et al. [64], we assign this fossil an age of 70.08 Ma. We applied a 95% prior age interval of 109.845 Ma based on the mean of the upper 95% credible intervals for fossil-based estimates of the age of Percomorpha [64]. Significantly, the age of the more commonly used calibration based on *Plectocretacicus* falls within this prior distribution. In a second set of analyses, we left the split between Lophiiformes and Tetraodontiformes uncalibrated based on perceived ambiguities in the interpretation of putative stem tetraodontiforms. We calibrated (7) the MRCA of African and neotropical cichlids using a minimum age of 46 Ma based on the stratigraphic information in Friedman et al. [64] with 95% of the prior age interval set at 85.625 Ma, which represents the mean of the upper 95% intervals for fossil-based estimates for Cichlidae [64]. This prior encompasses most [48] or all McMahan et al. [134] of the 95% HPD of ages estimates for the African and neotropical cichlid clade reported by recent molecular clock analyses that applied contrasting calibration strategies. Although this cichlid clade shows a geographic pattern congruent with Gondwanan vicariance, paleontological evidence based on both the distribution of cichlid bearing fossil horizons and the stratigraphic ages of closely related lineages reject an ancient origin for cichlids [64], as do fossil-calibrated timetrees that do not assume vicariance a priori [47],[48],[64],[134]. This finding reflects growing concerns that constraining ages based on present day distributions that reflect putative ancient vicariant events may bias our understanding of the evolutionary pathways that underlie the generation of modern biodiversity [135]. To assess the impact of the cichlid calibration on our divergence time estimates, we conducted a set of analyses leaving the divergence between African and neotropical cichlids uncalibrated.

### Profiling informativeness

To quantify phylogenetic informativeness (PI) for each dataset, site-specific rates and informativeness profiles were quantified using the program HyPhy in the PhyDesign web interface [136]. For site-rate calculations, we provided the consensus of the posterior distribution of trees resulting from the BEAST analyses and the respective mtDNA or nDNA alignments as inputs. In comparison to the consensus tree, using a subsample of trees from the posterior distribution inferred from each dataset yielded nearly identical PI profiles. PI plots were generated for the data partitions identified by PartitionFinder [106] used in the BEAST analyses, with PI profiles visualized in comparison to the corresponding consensus ultrametric tree. While removing data partitions in which the apex of the PI profile occurrs prior to the root of the tree would be an optimal strategy to limit the increased probability of partitions containing homoplasious site patterns [37], preliminary analyses suggested that this strategy was not feasible for the mitogenomic dataset as this required removal of almost all data. As the primary contention in age estimates between mtDNA and nDNA based analyses concerns whether or not the bulk of living actinopterygian diversity radiated during and after the Cretaceous, partitions whose profiles exhibit a decline from the peak of informativeness of greater than 5% prior to Jurassic-Cretaceous boundary were removed for subsequent analyses.

## Availability of supporting data

Xml files and associated tree files are available on Dryad: doi:10.5061/dryad.3rq51 and all sequence data is available on Genbank (http://www.ncbi.nlm.nih.gov/genbank).

## Competing interests

The authors declare that they have no competing interests.

## Authors’ contributions

All authors contributed to the overall design of this project. AD and JPT designed and conducted analysis of informativeness. AD, MF, and TJN designed and conducted analyses of molecular divergence times. AD was supported by all authors in the writing of this manuscript. All authors read and approved the final manuscript.

## Additional files

## Supplementary Material

Additional file 1: Table S1. Genbank accession numbers for fish nuclear gene dataset. **Table S2.** Genbank accession numbers for fish mitochondrial genome based dataset. Click here for file

Additional file 2: Figure S1. Comparison of mtDNA and nDNA chronograms for actinopterygians based on A) all data and B) analyses of excluding saturated partitions. Analyses utilized all calibrations except the cichlid calibration based on the interval of paleontological age estimates in Friedman et al. [64]. Bars indicate 95% HPD intervals of age estimates. Light bars indicate posterior probabilities greater than 0.95. Gray bars indicate posterior probabilities below 0.95. Phylogenetic informativeness profiles for both datasets are shown adjacent to the associated chronograms. Colours identify individual partitions. Click here for file

Additional file 3: Figure S2. Comparison of mtDNA and nDNA chronograms for actinopterygians based on A) all data and B) analyses of excluding saturated partitions. Analyses utilized all calibrations except the stem Tetraodontiform and cichlid calibration. Bars indicate 95% HPD intervals of age estimates. Light bars indicate posterior probabilities greater than 0.95. Gray bars indicate posterior probabilities below 0.95. Phylogenetic informativeness profiles for both datasets are shown adjacent to the associated chronograms. Colours identify individual partitions. Click here for file

Additional file 4: Figure S3. Visualizations of the Bayesian posterior density between replicate MCMC runs for selected parameters and different DNA datasets. Click here for file

## References

[B1] Martin W Gierl A Saedler H Molecular evidence for pre-Cretaceous angiosperm origins Nature 1989 339 46 48

[B2] Bell CD Soltis DE Soltis PS The age of the angiosperms: a molecular timescale without a clock Evolution 2005 59 6 1245 1258 16050101

[B3] Magallon S Using fossils to break long branches in molecular dating: a comparison of relaxed clocks applied to the origin of angiosperms Syst Biol 2010 59 4 384 399 20538759 10.1093/sysbio/syq027

[B4] Smith SA Beaulieu JM Donoghue MJ An uncorrelated relaxed-clock analysis suggests an earlier origin for flowering plants Proc Natl Acad Sci U S A 2010 107 13 5897 5902 20304790 10.1073/pnas.1001225107 PMC2851901

[B5] Meredith RW Janecka JE Gatesy J Ryder OA Fisher CA Teeling EC Goodbla A Eizirik E Simao TLL Stadler T Rabosky DL Honeycutt RL Flynn JJ Ingram CM Steiner C Williams TL Robinson TJ Burk-Herrick A Westerman M Ayoub NA Springer MS Murphy WJ Impacts of the Cretaceous Terrestrial Revolution and KPg extinction on mammal diversification Science 2011 334 6055 521 524 21940861 10.1126/science.1211028

[B6] Norman JE Ashley MV Phylogenetics of perissodactyla and tests of the molecular clock J Mol Evol 2000 50 1 11 21 10654255 10.1007/s002399910002

[B7] O'Leary MA Bloch JI Flynn JJ Gaudin TJ Giallombardo A Giannini NP Goldberg SL Kraatz BP Luo Z-X Meng J The placental mammal ancestor and the post–K-Pg radiation of placentals Science 2013 339 6120 662 667 23393258 10.1126/science.1229237

[B8] Springer MS Murphy WJ Eizirik E O'Brien SJ Placental mammal diversification and the Cretaceous-Tertiary boundary Proc Natl Acad Sci U S A 2003 100 3 1056 1061 12552136 10.1073/pnas.0334222100 PMC298725

[B9] Steiper ME Young NM Primate molecular divergence dates Mol Phylogenet Evol 2006 41 2 384 394 16815047 10.1016/j.ympev.2006.05.021

[B10] Theodor JM Molecular clock divergence estimates and the fossil record of Cetartiodactyla J Paleontol 2004 78 1 39 44

[B11] Dornburg A Santini F Alfaro ME The influence of model averaging on clade posteriors: an example using the triggerfishes (Family Balistidae) Syst Biol 2008 57 6 905 919 19085332 10.1080/10635150802562392

[B12] Hurley IA Mueller RL Dunn KA Schmidt EJ Friedman M Ho RK Prince VE Yang ZH Thomas MG Coates MI A new time-scale for ray-finned fish evolution Proc R Soc B 2007 274 1609 489 498 17476768 10.1098/rspb.2006.3749 PMC1766393

[B13] Near TJ Eytan RI Dornburg A Kuhn KL Moore JA Davis MP Wainwright PC Friedman M Smith WL Resolution of ray-finned fish phylogeny and timing of diversification Proc Natl Acad Sci U S A 2012 109 13698 13703 22869754 10.1073/pnas.1206625109 PMC3427055

[B14] Yamanoue Y Miya M Inoue JG Matsuura K Nishida M The mitochondrial genome of spotted green pufferfish *Tetraodon nigroviridis* (Teleostei: Tetraodontiformes) and divergence time estimation among model organisms in fishes Genes Genet Syst 2006 81 1 29 39 16607039 10.1266/ggs.81.29

[B15] Alfaro ME Santini F Brock CD Do reefs drive diversification in marine teleosts? Evidence from the pufferfish and their allies (Order Tetraodontiformes) Evolution 2007 61 9 2104 2126 17915358 10.1111/j.1558-5646.2007.00182.x

[B16] Pulquerio MJF Nichols RA Dates from the molecular clock: how wrong can we be? Trends Ecol Evol 2007 22 4 180 184 17157408 10.1016/j.tree.2006.11.013

[B17] Cooper A Fortey R Evolutionary explosions and the phylogenetic fuse Trends Ecol Evol 1998 13 4 151 156 21238236 10.1016/s0169-5347(97)01277-9

[B18] Dornburg A Beaulieu JM Oliver JC Near TJ Integrating fossil preservation biases in the selection of calibrations for molecular divergence time estimation Syst Biol 2011 60 4 519 527 21436104 10.1093/sysbio/syr019

[B19] Inoue J Donoghue PCJ Yang ZH The impact of the representation of fossil calibrations on Bayesian estimation of species divergence times Syst Biol 2010 59 1 74 89 20525621 10.1093/sysbio/syp078

[B20] Marshall CR A simple method for bracketing absolute divergence times on molecular phylogenies using multiple fossil calibration points Am Nat 2008 171 6 726 742 18462127 10.1086/587523

[B21] Ho SYW Phillips MJ Accounting for calibration uncertainty in phylogenetic estimation of evolutionary divergence times Syst Biol 2009 58 3 367 380 20525591 10.1093/sysbio/syp035

[B22] Marshall CR The fossil record and estimating divergence times between lineages: maximum divergence times and the importance of reliable phylogenies J Mol Evol 1990 30 400 408 2111853 10.1007/BF02101112

[B23] Benton MJ Donoghue PCJ Paleontological evidence to date the tree of life Mol Biol Evol 2007 24 1 26 53 17047029 10.1093/molbev/msl150

[B24] Benton MJ Donoghue PCJ Asher RJ Hedges SB, Kumar S Calibrating and constraining molecular clocks The Timetree of Life 2009 Oxford University Press, Oxford 35 86

[B25] Dornburg A Brandley MC McGowen MR Near TJ Relaxed clocks and inferences of heterogeneous patterns of nucleotide substitution and divergence time estimates across whales and dolphins (Mammalia: Cetacea) Mol Biol Evol 2012 29 2 721 736 21926070 10.1093/molbev/msr228

[B26] Soltis PS Soltis DE Savolainen V Crane PR Barraclough TG Rate heterogeneity among lineages of tracheophytes: integration of molecular and fossil data and evidence for molecular living fossils Proc Natl Acad Sci U S A 2002 99 7 4430 4435 11917101 10.1073/pnas.032087199 PMC123665

[B27] Drummond AJ Ho SYW Phillips MJ Rambaut A Relaxed phylogenetics and dating with confidence PLoS Biol 2006 4 5 699 710 10.1371/journal.pbio.0040088 PMC1395354 16683862

[B28] Ho SYW Larson G Molecular clocks: when times are a-changin' Trends Genet 2006 22 2 79 83 16356585 10.1016/j.tig.2005.11.006

[B29] Igawa T Kurabayashi A Usuki C Fujii T Sumida M Complete mitochondrial genomes of three neobatrachian anurans: a case study of divergence time estimation using different data and calibration settings Gene 2008 407 1 116 129 17997052 10.1016/j.gene.2007.10.001

[B30] Brandley MC Wang Y Guo X Nieto Montes De Oca A Feria-Ortiz M Hikida T Ota H Accommodating heterogenous rates of evolution in molecular divergence dating methods: an example using intercontinental dispersal of *Plestiodon* (*Eumeces*) lizards Syst Biol 2011 60 3 15 20952756 10.1093/sysbio/syq045

[B31] Xia XH Xie Z Salemi M Chen L Wang Y An index of substitution saturation and its application Mol Phylogenet Evol 2003 26 1 1 7 12470932 10.1016/s1055-7903(02)00326-3

[B32] Yang Z Among-site rate variation and its impact on phylogenetic analyses Trends Ecol Evol 1996 11 367 372 21237881 10.1016/0169-5347(96)10041-0

[B33] Graybeal A Evaluating the phylogenetic utility of genes: a search for genes informative about deep divergences among vertebrates Syst Biol 1994 43 2 174 193

[B34] Moritz C Schneider CJ Wake DB Evolutionary relationships within the *Ensatina eschscholtzii* complex confirm the ring species interpretation Syst Biol 1992 41 273 291

[B35] Xia X, Lemey P: **Assessing Substitution Saturation With DAMBE**. In *The Phylogenetic Handbook: a Practical Approach to Phylogenetic Analysis and Hypothesis Testing.* Edited by Philippe L, Marco S, Anne-Mieke V. Cambridge University Press; 2009:611–626.

[B36] Townsend JP Profiling phylogenetic informativeness Syst Biol 2007 56 2 222 231 17464879 10.1080/10635150701311362

[B37] Townsend JP Leuenberger C Taxon sampling and the optimal rates of evolution for phylogenetic inference Syst Biol 2011 60 358 365 21303824 10.1093/sysbio/syq097

[B38] Azuma Y Kumazawa Y Miya M Mabuchi K Nishida M Mitogenomic evaluation of the historical biogeography of cichlids toward reliable dating of teleostean divergences BMC Evol Biol 2008 8 1 215 18651942 10.1186/1471-2148-8-215 PMC2496912

[B39] Zhang P Wake DB Higher-level salamander relationships and divergence dates inferred from complete mitochondrial genomes Mol Phylogenet Evol 2009 53 2 492 508 19595776 10.1016/j.ympev.2009.07.010

[B40] Miya M Pietsch TW Orr JW Arnold RJ Satoh TP Shedlock AM Ho HC Shimazaki M Yabe M Nishida M Evolutionary history of anglerfishes (Teleostei: Lophiiformes): a mitogenomic perspective BMC Evol Biol 2010 10 1 58 20178642 10.1186/1471-2148-10-58 PMC2836326

[B41] Yamanoue Y Miya M Doi H Mabuchi K Sakai H Nishida M Multiple invasions into freshwater by pufferfishes (teleostei: tetraodontidae): a mitogenomic perspective Plos One 2011 6 2 e17410 21364898 10.1371/journal.pone.0017410 PMC3045446

[B42] Zheng Y Peng R Kuro-O M Zeng X Exploring patterns and extent of bias in estimating divergence time from mitochondrial DNA sequence data in a particular lineage: a case study of salamanders (Order Caudata) Mol Biol Evol 2011 28 9 2521 2535 21422243 10.1093/molbev/msr072

[B43] Gardiner BG The relationships of the palaeoniscid fishes, a review based on new specimens of *Mimia* and *Moythomasia* from Upper Devonian of Western Australia Bull Brit Mus (Nat Hist) Geol 1984 37 4 173 428

[B44] Friedman M Sallan LC Five hundred million years of extinction and recovery: a Phanerozoic survey of large-scale diversity patterns in fishes Palaeontology 2012 55 707 742

[B45] Nelson JS Fishes of the World, 4th Edition 2006 John Wiley, Hoboken

[B46] Alfaro ME Santini F Brock C Alamillo H Dornburg A Rabosky DL Carnevale G Harmon LJ Nine exceptional radiations plus high turnover explain species diversity in jawed vertebrates Proc Natl Acad Sci U S A 2009 106 32 13410 13414 19633192 10.1073/pnas.0811087106 PMC2715324

[B47] Betancur-R R, Broughton RE, Wiley EO, Carpenter K, López JA, Li C, Holcroft NI, Arcila D, Sanciangco M, Cureton JC II: **The tree of life and a new classification of bony fishes.** *PLoS Currents* 2013, **5:**. 10.1371/currents.tol.53ba26640df0ccaee75bb165c8c26288 PMC3644299 23653398

[B48] Near TJ Dornburg A Eytan RI Keck BP Smith WL Kuhn KL Moore JA Price SA Burbrink FT Friedman M Phylogeny and tempo of diversification in the superradiation of spiny-rayed fishes Proc Natl Acad Sci 2013 110 31 12738 12743 23858462 10.1073/pnas.1304661110 PMC3732986

[B49] Santini F Harmon LJ Carnevale G Alfaro ME Did genome duplication drive the origin of teleosts? A comparative study of diversification in ray-finned fishes BMC Evol Biol 2009 9 164 19664233 10.1186/1471-2148-9-194 PMC2743667

[B50] Miya M Friedman M Satoh TP Takeshima H Sado T Iwasaki W Yamanoue Y Nakatani M Mabuchi K Inoue JG Evolutionary origin of the scombridae (tunas and mackerels): members of a paleogene adaptive radiation with 14 other pelagic fish families PLoS One 2013 8 9 e73535 24023883 10.1371/journal.pone.0073535 PMC3762723

[B51] Blieck A From adaptive radiations to biotic crises in Palaeozoic vertebrates: a geobiological approach Geologica Belgica 2011 14 3–4 203 227

[B52] Friedman M Explosive morphological diversification of spiny-finned teleost fishes in the aftermath of the end-Cretaceous extinction Proc R Soc B 2010 277 1688 1675 1683 20133356 10.1098/rspb.2009.2177 PMC2871855

[B53] Lloyd GT Friedman M A survey of palaeontological sampling biases in fishes based on the phanerozoic record of Great Britain Palaeogeogr Palaeoclimat Palaecol 2012 372 5 17

[B54] Cavin L Forey PL Using ghost lineages to identify diversification events in the fossil record Biol Lett 2007 3 2 201 204 17284405 10.1098/rsbl.2006.0602 PMC2375936

[B55] Cavin L Forey PL Lecuyer C Correlation between environment and late mesozoic ray-finned fish evolution Palaeogeogr Palaeoclimat Palaecol 2007 245 3–4 353 367

[B56] Broughton RE Broughton RE Nelson JS, Schultze H-P, Schultze H-P, Wilson MVH Phylogeny of Teleosts Based on Mitochondrial Sequences Origin and Phylogenetic Interrelationships of Teleosts 2010 Verlag Dr. Friedrich Pfeil, Munchen 61 76

[B57] Inoue JG Miya M Venkatesh B Nishida M The mitochondrial genome of Indonesian coelacanth *Latimeria menadoensis* (Sarcopterygii: Coelacanthiformes) and divergence time estimation between the two coelacanths Gene 2005 349 227 235 15777665 10.1016/j.gene.2005.01.008

[B58] Near TJ Dornburg A Tokita M Suzuki D Brandley MC Friedman M Boom and bust: ancient and recent diversification in bichirs (polypteridae: actinopterygii), a relictual lineage of ray‐finned fishes Evolution 2013 68 4 1014 1026 24274466 10.1111/evo.12323

[B59] Phillips MJ Branch-length estimation bias misleads molecular dating for a vertebrate mitochondrial phylogeny Gene 2009 441 1 132 140 18809474 10.1016/j.gene.2008.08.017

[B60] Sallan LC Friedman M Heads or tails: staged diversification in vertebrate evolutionary radiations Proc R Soc B 2012 279 1735 2025 2032 22189401 10.1098/rspb.2011.2454 PMC3311904

[B61] Dornburg A Sidlauskas B Santini F Sorenson L Near TJ Alfaro ME The influence of an innovative locomotor strategy on the phenotypic diversification of triggerfish (family: balistidae) Evolution 2011 65 7 1912 1926 21729047 10.1111/j.1558-5646.2011.01275.x

[B62] Santini F Sorenson L Alfaro ME A new multi-locus timescale reveals the evolutionary basis of diversity patterns in triggerfishes and filefishes (Balistidae, Monacanthidae; Tetraodontiformes) Mol Phylogenet Evol 2013 69 1 165 176 23727054 10.1016/j.ympev.2013.05.015

[B63] Santini F Sorenson L Marcroft T Dornburg A Alfaro ME A multilocus molecular phylogeny of boxfishes (Aracanidae, Ostraciidae; Tetraodontiformes) Mol Phylogenet Evol 2013 66 1 153 160 23036494 10.1016/j.ympev.2012.09.022

[B64] Friedman M Keck BP Dornburg A Eytan RI Martin CH Hulsey CD Wainwright PC Near TJ Molecular and fossil evidence place the origin of cichlid fishes long after Gondwanan rifting Proceedings of the Royal Society B: Biological Sciences 2013 280 1770 20131733 24048155 10.1098/rspb.2013.1733 PMC3779330

[B65] López Fernández H Arbour JH Winemiller K Honeycutt RL Testing for ancient adaptive radiations in Neotropical cichlid fishes Evolution 2013 67 5 1321 1337 23617911 10.1111/evo.12038

[B66] Matschiner M Hanel R Salzburger W On the origin and trigger of the notothenioid adaptive radiation Plos One 2011 6 4 e18911 21533117 10.1371/journal.pone.0018911 PMC3078932

[B67] Near TJ Dornburg A Kuhn KL Eastman JT Pennington JN Patarnello T Zane L Fernandez DA Jones CD Ancient climate change, antifreeze, and the evolutionary diversification of Antarctic fishes Proc Natl Acad Sci U S A 2012 109 9 3434 3439 22331888 10.1073/pnas.1115169109 PMC3295276

[B68] Graybeal A The phylogenetic utility of cytochrome *b*: lessons from bufonid frogs Mol Phylogenet Evol 1993 2 256 269 8136925 10.1006/mpev.1993.1024

[B69] Blouin MS Yowell CA Courtney CH Dame JB Substitution bias, rapid saturation, and the use of mtDNA for nematode systematics Mol Biol Evol 1998 15 12 1719 1727 9866206 10.1093/oxfordjournals.molbev.a025898

[B70] Pratt RC Gibb GC Morgan-Richards M Phillips MJ Hendy MD Penny D Toward resolving deep Neoaves phylogeny: data, signal enhancement, and priors Mol Biol Evol 2009 26 2 313 326 18981298 10.1093/molbev/msn248

[B71] Phillips MJ Penny D The root of the mammalian tree inferred from whole mitochondrial genomes Mol Phylogenet Evol 2003 28 2 171 185 12878457 10.1016/s1055-7903(03)00057-5

[B72] Honeycutt RL Adkins RM Higher level systematics of eutherian mammals: an assessment of molecular characters and phylogenetic hypotheses Annu Rev Ecol Syst 1993 24 279 305

[B73] Jeffroy O Brinkmann H Delsuc F Philippe H Phylogenomics: the beginning of incongruence? Trends Genet 2006 22 4 225 231 16490279 10.1016/j.tig.2006.02.003

[B74] Morgan-Richards M Trewick SA Bartosch-Härlid A Kardailsky O Phillips MJ McLenachan PA Penny D Bird evolution: testing the Metaves clade with six new mitochondrial genomes BMC Evol Biol 2008 8 1 20 18215323 10.1186/1471-2148-8-20 PMC2259304

[B75] Townsend JP Lopez-Giraldez F Optimal selection of gene and ingroup taxon sampling for resolving phylogenetic relationships Syst Biol 2010 59 4 446 457 20547780 10.1093/sysbio/syq025

[B76] Townsend JP Lopez-Giraldez F Friedman R The phylogenetic informativeness of nucleotide and amino acid sequences for reconstructing the vertebrate tree J Mol Evol 2008 67 5 437 447 18696029 10.1007/s00239-008-9142-0

[B77] Townsend JP Su Z Tekle YI Phylogenetic signal and noise: predicting the power of a data set to resolve phylogeny Syst Biol 2012 61 5 835 849 22389443 10.1093/sysbio/sys036

[B78] Doyle JA Donoghue MJ Phylogenies and angiosperm diversification Paleobio 1993 19 2 141 167

[B79] Near TJ Meylan PA Shaffer HB Assessing concordance of fossil calibration points in molecular clock studies: an example using turtles Am Nat 2005 165 2 137 146 15729646 10.1086/427734

[B80] Pyron RA A likelihood method for assessing molecular divergence time estimates and the placement of fossil calibrations Syst Biol 2010 59 2 185 194 20525629 10.1093/sysbio/syp090

[B81] Rutschmann F Eriksson T Abu Salim K Conti E Assessing calibration uncertainty in molecular dating: the assignment of fossils to alternative calibration points Syst Biol 2007 56 4 591 608 17654364 10.1080/10635150701491156

[B82] Drummond AJ Suchard MA Bayesian random local clocks, or one rate to rule them all BMC Biol 2010 8 114 20807414 10.1186/1741-7007-8-114 PMC2949620

[B83] Aris-Brosou S Yang ZH Effects of models of rate evolution on estimation of divergence dates with special reference to the metazoan 18S ribosomal RNA Phylogeny Syst Biol 2002 51 5 703 714 12396585 10.1080/10635150290102375

[B84] Lepage T Bryant D Philippe H Lartillot N A general comparison of relaxed molecular clock models Mol Biol Evol 2007 24 12 2669 2680 17890241 10.1093/molbev/msm193

[B85] Yoder AD Yang Z Estimation of primate speciation dates using local molecular clocks Mol Biol Evol 2000 17 7 1081 1090 10889221 10.1093/oxfordjournals.molbev.a026389

[B86] Brown WM George M Wilson AC Rapid evolution of animal mitochondrial DNA Proc Natl Acad Sci 1979 76 4 1967 1971 109836 10.1073/pnas.76.4.1967 PMC383514

[B87] Jiang ZJ Castoe TA Austin CC Burbrink FT Herron MD McGuire JA Parkinson CL Pollock DD Comparative mitochondrial genomics of snakes: extraordinary substitution rate dynamics and functionality of the duplicate control region BMC Evol Biol 2007 7 1 123 17655768 10.1186/1471-2148-7-123 PMC1950710

[B88] Wolfe KH Li W-H Sharp PM Rates of nucleotide substitution vary greatly among plant mitochondrial, chloroplast, and nuclear DNAs Proc Natl Acad Sci 1987 84 24 9054 9058 3480529 10.1073/pnas.84.24.9054 PMC299690

[B89] Smith DR Arrigo KR Alderkamp A-C Allen AE Massive difference in synonymous substitution rates among mitochondrial, plastid, and nuclear genes of < i > Phaeocystis</i > algae Mol Phylogenet Evol 2014 71 36 40 24216019 10.1016/j.ympev.2013.10.018

[B90] Shearer T Van Oppen M Romano S Wörheide G Slow mitochondrial DNA sequence evolution in the Anthozoa (Cnidaria) Mol Ecol 2002 11 12 2475 2487 12453233 10.1046/j.1365-294x.2002.01652.x

[B91] Hellberg ME No variation and low synonymous substitution rates in coral mtDNA despite high nuclear variation BMC Evol Biol 2006 6 1 24 16542456 10.1186/1471-2148-6-24 PMC1431588

[B92] Chen I-P Tang C-Y Chiou C-Y Hsu J-H Wei NV Wallace CC Muir P Wu H Chen CA Comparative analyses of coding and noncoding DNA regions indicate that Acropora (Anthozoa: Scleractina) possesses a similar evolutionary tempo of nuclear vs. mitochondrial genomes as in plants Marine Biotechnol 2009 11 1 141 152 10.1007/s10126-008-9129-2 18670809

[B93] Dávalos LM Perkins SL Saturation and base composition bias explain phylogenomic conflict in < i > Plasmodium</i> Genomics 2008 91 5 433 442 18313259 10.1016/j.ygeno.2008.01.006

[B94] Pick K Philippe H Schreiber F Erpenbeck D Jackson D Wrede P Wiens M Alié A Morgenstern B Manuel M Improved phylogenomic taxon sampling noticeably affects nonbilaterian relationships Mol Biol Evol 2010 27 9 1983 1987 20378579 10.1093/molbev/msq089 PMC2922619

[B95] Chiari Y Cahais V Galtier N Delsuc F Phylogenomic analyses support the position of turtles as the sister group of birds and crocodiles (Archosauria) BMC Biol 2012 10 1 65 22839781 10.1186/1741-7007-10-65 PMC3473239

[B96] Parks M Cronn R Liston A Separating the wheat from the chaff: mitigating the effects of noise in a plastome phylogenomic data set from Pinus L. (Pinaceae) BMC Evol Biol 2012 12 1 100 22731878 10.1186/1471-2148-12-100 PMC3475122

[B97] Lemmon AR Emme SA Lemmon EM Anchored hybrid enrichment for massively high-throughput phylogenomics Syst Biol 2012 61 5 727 744 22605266 10.1093/sysbio/sys049

[B98] Faircloth BC, Chang J, Alfaro ME: **TAPIR enables high-throughput estimation and comparison of phylogenetic informativeness using locus-specific substitution models.** *arXiv preprint arXiv:12021215* 2012, 1215.

[B99] Philippe H Brinkmann H Lavrov DV Littlewood DTJ Manuel M Worheide G Baurain D Resolving difficult phylogenetic auestions: why more sequences are not enough PLoS Biol 2011 9 3 e1000602 21423652 10.1371/journal.pbio.1000602 PMC3057953

[B100] Dunn CW Hejnol A Matus DQ Pang K Browne WE Smith SA Seaver E Rouse GW Obst M Edgecombe GD Broad phylogenomic sampling improves resolution of the animal tree of life Nature 2008 452 7188 745 749 18322464 10.1038/nature06614

[B101] Delsuc F Brinkmann H Philippe H Phylogenomics and the reconstruction of the tree of life Nature Rev Genet 2005 6 5 361 375 15861208 10.1038/nrg1603

[B102] Romiguier J, Ranwez V, Delsuc F, Galtier N, Douzery EJ: **Less is more in mammalian phylogenomics: AT-rich genes minimize tree conflicts and unravel the root of placental mammals.** *Mol Biol Evol* 2013. mst116. 10.1093/molbev/mst116 23813978

[B103] Lin J Chen G Gu L Shen Y Zheng M Zheng W Hu X Zhang X Qiu Y Liu X Phylogenetic affinity of tree shrews to Glires is attributed to fast evolution rate Mol Phylogenet Evol 2014 71 193 200 24333622 10.1016/j.ympev.2013.12.001

[B104] Edgar RC MUSCLE: multiple sequence alignment with high accuracy and high throughput Nucleic Acids Res 2004 32 5 1792 1797 15034147 10.1093/nar/gkh340 PMC390337

[B105] Smith SA Dunn CW Phyutility: a phyloinformatics tool for trees, alignments and molecular data Bioinformatics 2008 24 5 715 716 18227120 10.1093/bioinformatics/btm619

[B106] Lanfear R Calcott B Ho SYW Guindon S PartitionFinder: combined selection of partitioning schemes and substitution models for phylogenetic analyses Mol Biol Evol 2012 29 6 1695 1701 22319168 10.1093/molbev/mss020

[B107] Drummond AJ Rambaut A BEAST: Bayesian evolutionary analysis by sampling trees BMC Evol Biol 2007 7 214 17996036 10.1186/1471-2148-7-214 PMC2247476

[B108] http://tree.bio.ed.ac.uk/software/tracer Rambaut A, Drummond AJ: **Tracer, MCMC Trace Analysis Package.** 15th edition. 2003. Available from .

[B109] Coates MI Actinopterygians from the Namurian of Bearsden, Scotland, with comments on early actinopterygian neurocrania Zool J Linn Soc 1998 122 1–2 27 59

[B110] Li CH Lu GQ Ortí G Optimal data partitioning and a test case for ray-finned fishes (Actinopterygii) based on ten nuclear loci Syst Biol 2008 57 4 519 539 18622808 10.1080/10635150802206883

[B111] Gardiner BG Schaeffer B Interrelationships of lower actinopterygian fishes Zool J Linn Soc 1989 97 135 187

[B112] Miya M Takeshima H Endo H Ishiguro NB Inoue JG Mukai T Satoh TP Yamaguchi M Kawaguchi A Mabuchi K Shirai SM Nishida M Major patterns of higher teleostean phylogenies: a new perspective based on 100 complete mitochondrial DNA sequences Mol Phylogenet Evol 2003 26 121 138 12470944 10.1016/s1055-7903(02)00332-9

[B113] Johnson GD Patterson C Percomorph phylogeny: a survey of acanthomorphs and a new proposal Bull Mar Sci 1993 52 1 554 626

[B114] Warnock RCM Yang ZH Donoghue PCJ Exploring uncertainty in the calibration of the molecular clock Biol Lett 2012 8 1 156 159 21865245 10.1098/rsbl.2011.0710 PMC3259980

[B115] Morrow JR Sandberg CA Evolution of Devonian carbonate-shelf margin Nevada Geosphere 2008 4 2 445 458

[B116] Zhu M Zhao WJ Jia LT Lu J Qiao T Qu QM The oldest articulated osteichthyan reveals mosaic gnathostome characters Nature 2009 458 7237 469 474 19325627 10.1038/nature07855

[B117] Dineley DL Metcalf SJ Fossil Fishes of Great Britain 1999 Peterborough, Joint Nature Conservation Committee

[B118] Menning M Weyer D Drozdzewski G Van Amerom HWJ Wendt I A Carboniferous timescale 2000: discussion and use of geological parameters as time indicators from central and western Europe Geol Jahrbuch 2000 2000 A156 3 44

[B119] Coates MI Endocranial preservation of a Carboniferous actinopterygian from Lancashire, UK, and the interrelationships of primitive actinopterygians Phil Trans R Soc B 1999 354 1382 435 462

[B120] Xu G-H, Gao K-Q, Finarelli J, Xu G-H, Gao K-Q, Finarelli J: **A revision of the Middle Triassic scanilepiform fish Fukangichthys longidorsalis from Xinjiang, China, with comments on the phylogeny of the Actinopteri.** *J Vert Paleo*. in press.

[B121] Gradstein FM Ogg G Schmitz M The Geologic Time Scale 2012 2-Volume Set 2012 Elsevier, Amsterdam

[B122] Olsen PE The skull and pectoral girdle of the parasemionotid fish *Watsonulus eugnathoides* from the early Triassic Sakamena group of Madagascar, with comments on the relationships of the holostean fishes J Vert Paleo 1984 4 481 499

[B123] Catuneanu O Wopfer H Eriksson PG Carincross B Rubidge BS Smith RMH Hancox PJ The Karoo basins of South-Central Africa J Afr Earth Sci 2005 43 211 253

[B124] Ogg JG Ogg JG Gradstein F, Ogg J, Smith A The Triassic Period A Geologic Time Scale 2004 Cambridge University Press, Cambridge 271 306

[B125] Xu GH Gao KQ A new scanilepiform from the lower triassic of northern Gansu Province, China, and phylogenetic relationships of non-teleostean actinopterygii Zool J Linn Soc 2011 161 3 595 612

[B126] Patterson C A review of Mesozoic acanthopterygian fishes, with special reference to those of the English Chalk Phil Trans R Soc B 1964 247 739 213 482

[B127] Rosen DE Greenwood PH, Miles RS, Patterson C, Greenwood PH, Miles RS, Patterson C Interrelationships of Higher Euteleostean Fishes Interrelationships of Fishes 1973 Academic Press, London 397 513

[B128] Santini F Tyler JC A phylogeny of the families of fossil and extant tetraodontiform fishes (Acanthomorpha, Tetraodontiformes), upper Cretaceous to recent Zool J Linn Soc 2003 139 4 565 617

[B129] Gallo V Carvalho MSSD Souto AA A possible occurrence of Diodontidae (Teleostei, Tetraodontiformes) in the Upper Cretaceous of the Paraíba Basin, Northeastern Brazil Cretaceous Research 2009 30 3 599 604

[B130] Yang ZH Rannala B Bayesian estimation of species divergence times under a molecular clock using multiple fossil calibrations with soft bounds Mol Biol Evol 2006 23 1 212 226 16177230 10.1093/molbev/msj024

[B131] Sorbini L Segnalazione di un plettognato Cretacico Plectocretacicus nov General Boll Mus Civ Stor Nat Verona 1979 6 1 4

[B132] Tyler JC, Sorbini L: *New Superfamily and Three new Families of Tetraodontiform Fishes from the Upper Cretaceous: The Earliest and Most Morphologically Primitive Plectognaths.* 1996.

[B133] González-Rodríguez KA Schultze H-P Arratia G Arratia G, Arratia G, Schultze H-P, Schultze H-P, Wilson MVH Minature Armored Acanthomorph Teleosts from the Albian/Cenomanian (Cretaceous) of Mexico Mesozoic Fishes 5—Global Diversity and Evolution 2013 Verlag Dr Friedrich Pfeil, Munich 457 487

[B134] McMahan CD Chakrabarty P Sparks JS Smith WL Davis MP Temporal patterns of diversification across global cichlid biodiversity (Acanthomorpha: Cichlidae) PLoS One 2013 8 8 e71162 23990936 10.1371/journal.pone.0071162 PMC3747193

[B135] Waters JM Trewick SA Paterson AM Spencer HG Kennedy M Craw D Burridge CP Wallis GP Biogeography off the tracks Syst Biol 2013 62 3 494 498 23427288 10.1093/sysbio/syt013

[B136] Lopez-Giraldez F Townsend JP PhyDesign: an online application for profiling phylogenetic informativeness BMC Evol Biol 2011 11 152 21627831 10.1186/1471-2148-11-152 PMC3124428

