# Supplementary material for: Phylogenetic informativeness reconciles ray-finned fish molecular divergence times
Source: BMC Evol Biol. 2014 Aug 8;14:169. doi: 10.1186/s12862-014-0169-0 (PMC4236503; doi:10.1186/s12862-014-0169-0)
Supplement: Additional file 1: Table S1. — Genbank accession numbers for fish nuclear gene dataset. Table S2. Genbank accession numbers for fish mitochondrial genome based dataset. [file s12862-014-0169-0-S1.doc]

| Supplemental Table 1: Genbank accession numbers for fish nuclear gene dataset | | | | | | | | |
| --- | --- | --- | --- | --- | --- | --- | --- | --- |
| *Taxon* | Glyt | myh6 | plag12 | Ptr | rag1 | SH3PX3 | tbr1 | zic1 |
| *Albula vulpes* | JX190390 | NA | JX190653 | JX190803 | NA | NA | JX191136 | JX191247 |
| *Alosa pseudoharengus* | GU368586 | JX190550 | JX190674 | DQ912115 | JX190936 | JX191065 | NA | JX191263 |
| *Ameiurus natalis* | JX190416 | JX190561 | JX190685 | JX190818 | JX190945 | JX191072 | JX191151 | NA |
| *Amia calva* | JX190389 | JX190531 | JX190652 | JX190802 | JX190919 | JX191055 | EF032961 | JX191246 |
| *Anguilla rostrata* | JX190395 | NA | JX190659 | NA | JX190924 | NA | NA | JX191252 |
| *Aphredoderus sayanus* | JX190462 | JX190586 | JX190723 | JX190850 | NA | NA | JX191187 | JX191311 |
| *Apteronotus albifrons* | NA | JX190554 | JX190678 | JX190812 | JX190940 | NA | NA | JX191267 |
| *Arapaima gigas* | NA | NA | JX190708 | NA | JX190970 | NA | NA | NA |
| *Argentina silus* | JX190423 | NA | JX190692 | JX190822 | JX190952 | NA | JX191158 | JX191275 |
| *Atractosteus spatula* | JN853399 | JN853567 | NA | NA | NA | JN853507 | JN853433 | JN853469 |
| *Bothus lunatus* | JX190501 | NA | JX190769 | JX190891 | JX191026 | NA | NA | NA |
| *Chanos chanos* | JX190408 | JX190551 | JX190675 | JX190809 | JX190937 | JX191066 | JX191147 | JX191264 |
| *Coryphaenoides rupestris* | EU001915 | NA | EU001969 | FJ215233 | EU002077 | NA | NA | NA |
| *Danio rerio* | Ensembl | Ensembl | Ensembl | Ensembl | Ensembl | Ensembl | Ensembl | Ensembl |
| *Diodon holocanthus* | JX189774 | JX189455 | JX190239 | JX189934 | JX189526 | JX190070 | JX189307 | JX189159 |
| *Elops saurus* | JX190394 | JX190535 | JX190657 | FJ896408 | JX190922 | JX191056 | JX191139 | JX191250 |
| *Esox lucius* | JX190428 | EU002099 | JX190695 | JX190825 | EU002069 | JX191074 | JX191163 | EU001870 |
| *Gadus morhua* | EU001906 | EU002100 | EU001960 | NA | EU002071 | EU002126 | NA | EU001871 |
| *Gambusia affinis* | JX189643 | JX189326 | JX190090 | JX189797 | JX189550 | JX189953 | JX189179 | JX189027 |
| *Gasterosteus aculeatus* | JX189649 | JX189331 | JX190096 | JX189803 | JX189556 | JX189959 | JX189183 | JX189032 |
| *Heros appendiculatus* | JQ352850 | JQ352895 | NA | JQ353049 | NA | JQ353118 | NA | JQ353158 |
| *Hiodon alosoides* | NA | JX190543 | JX190670 | AY430200 | JX190930 | JX191058 | JX191142 | JX191257 |
| *Hucho perryi* | JX190442 | JX190569 | JX190702 | JX190832 | JX190964 | JX191081 | JX191172 | JX191290 |
| *Lophius americanus* | JX189629 | JX189314 | JX190076 | JX189781 | JX189533 | JX189940 | JX189166 | JX189012 |
| *Lycodes terraenovae* | EF032931 | EF033022 | EF032957 | NA | EF033009 | EF033035 | EF032970 | EF032918 |
| *Monopterus albus* | JQ352812 | JQ352857 | JX190098 | JQ353018 | JX189557 | JQ353087 | JX189185 | JQ353124 |
| *Myripristis violacea* | JX190474 | JX190597 | JX190735 | JX190861 | NA | JX191091 | JX191193 | JX191320 |
| *Neoscopelus microchir* | JX190456 | JX190581 | JX190715 | NA | JX190976 | NA | JX191182 | JX191303 |
| *Oryzias latipes* | Ensembl | Ensembl | Ensembl | Ensembl | Ensembl | Ensembl | Ensembl | Ensembl |
| *Ospariichthys uncirostris* | JX190417 | JX190562 | JX190686 | JX190819 | JX190946 | NA | JX191152 | JX191270 |
| *Polymixia japonica* | JX189626 | JX189311 | JX190073 | JX189778 | JX189530 | JX189938 | JX189164 | JX189010 |
| *Polyodon spathula* | NA | JX190530 | JX190651 | JX190801 | JX190918 | JX191054 | NA | JX191245 |
| *Polypterus ornatipinnis* | NA | JX190528 | JX190649 | JX190799 | NA | JX191052 | NA | JX191243 |
| *Porichthys notatus* | JX190480 | JX190603 | JX190741 | JX190867 | JX191000 | JX191097 | JX191199 | JX191325 |
| *Sebastes fasciatus* | JX189655 | JX189337 | JX190103 | JX189809 | JX189562 | NA | JX189190 | JX189038 |
| *Stomias boa* | EU001914 | NA | EU001968 | NA | EU002076 | EU002134 | EU001995 | EU001879 |
| *Synodus foetens* | JX190455 | NA | JX190714 | JX190844 | JX190975 | JX191084 | JX191181 | JX191302 |
| *Tetraodon miurus* | JX190516 | JX190639 | JX190784 | JX190905 | JX191040 | JX191126 | JX191231 | JX191358 |
| *Zeus faber* | JX190467 | JX190590 | JX190728 | JX190854 | JX190988 | JX191088 | JX191189 | NA |

| Supplemental Table 2: Genbank accession numbers for fish mitochondrial genome based dataset | | | |
| --- | --- | --- | --- |
| Taxon | Accession | Taxon | Accession |
| *Amia calva* | NC 004742 | *Lycodes toyamensis* | NC 004409 |
| *Anguilla rostrata* | NC 006531 | *Monopterus albus* | NC 003192 |
| *Aphredoderus sayanus* | NC 004372 | *Mugil cephalus* | NC 003182 |
| *Apteronotus albifrons* | NC 004692 | *Myripristis berndti* | NC 003189 |
| *Carassius auratus* | NC 002079 | *Neoscopelus microchir* | NC 003180 |
| *Chanos chanos* | NC 004693 | *Notropis stramineus* | NC 008110 |
| *Chauliodus sloani* | NC 003159 | *Oncorhynchus mykiss* | NC 001717 |
| *Diodon holocanthus* | NC 009866 | *Oreochromis mossambicus* | NC 007231 |
| *Dorosoma cepedianum* | NC 008107 | *Oryzias latipes* | NC 004387 |
| *Elops saurus* | NC 005803 | *Osteoglossum bicirrhosum* | NC 003095 |
| *Esox lucius* | NC 004593 | *Paralichthys olivaceus* | NC 002386 |
| *Fundulus olivaceus* | NC 011380 | *Polymixia lowei* | NC 003181 |
| *Gadus morhua* | NC 002081 | *Polyodon spathula* | NC 004419 |
| *Gambusia affinis* | NC 004388 | *Polypterus ornatipinnis* | NC 001778 |
| *Gasterosteus aculeatus* | NC 003174 | *Porichthys myriaster* | NC 006920 |
| *Glossanodon semifasciatus* | NC 004595 | *Sebastes schlegeli* | NC 005450 |
| *Hiodon alosoides* | NC 005145 | *Synodus variegatus* | NC 007228 |
| *Hypselecara temporalis* | NC 011168 | *Takifugu rubripes* | NC 004299 |
| *Ictalurus punctatus* | NC 003489 | *Tetraodon nigroviridis* | NC 007176 |
| *Lepisosteus osseus* | NC 008104 | *Ventrifossa garmani* | NC 008225 |
| *Lophius americanus* | NC 004380 | *Zeus faber* | NC 003190 |
| *Lutjanus bengalensis* | NC 011275 |  |  |
|  | | | |
|  | | | |
