# Supplementary figures and images for: Phylogenetic informativeness reconciles ray-finned fish molecular divergence times
Source: BMC Evol Biol. 2014 Aug 8;14:169. doi: 10.1186/s12862-014-0169-0 (PMC4236503; doi:10.1186/s12862-014-0169-0)

A.

Nuclear Genes

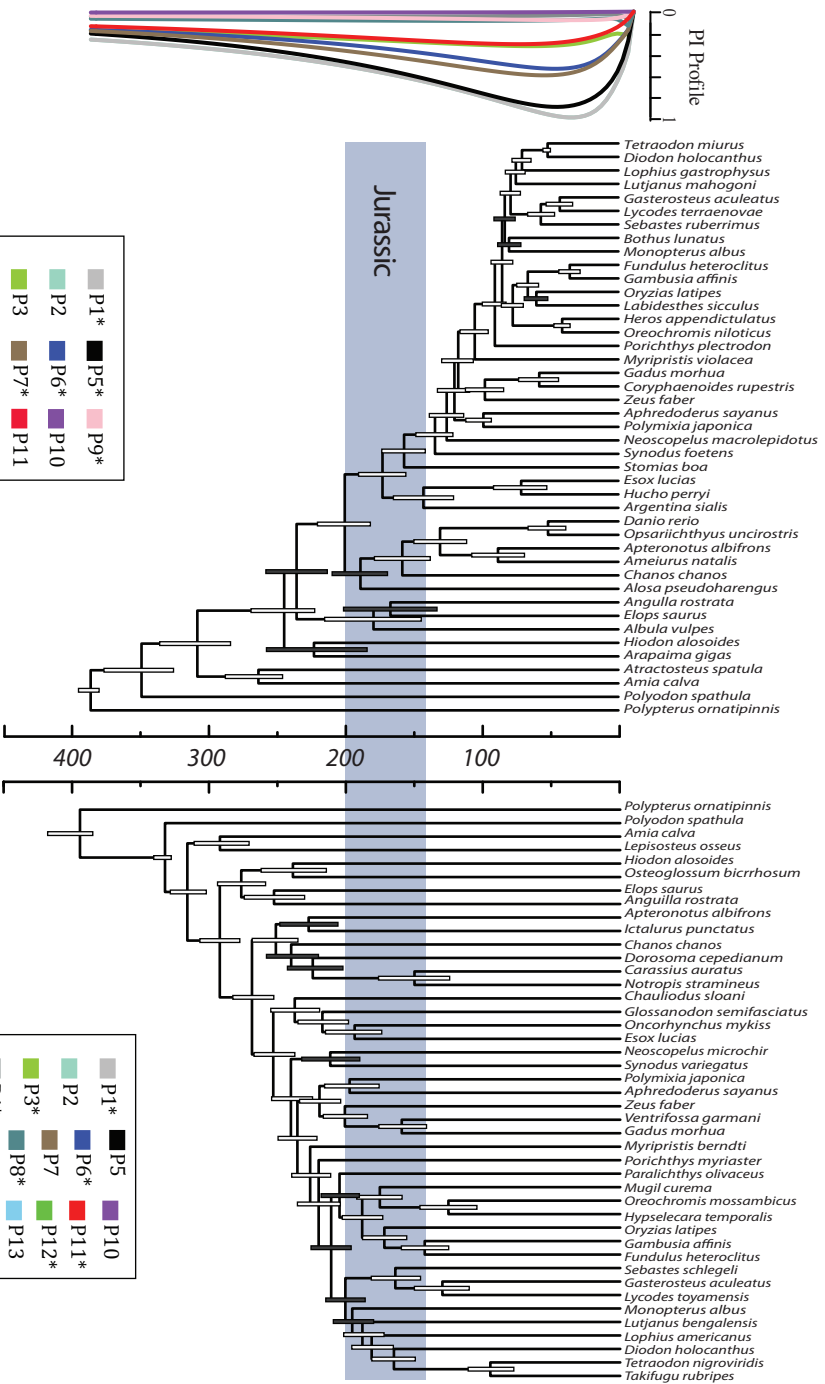

Mitochondrial Genes

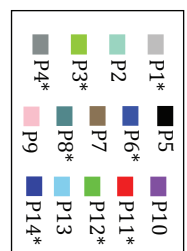

B.

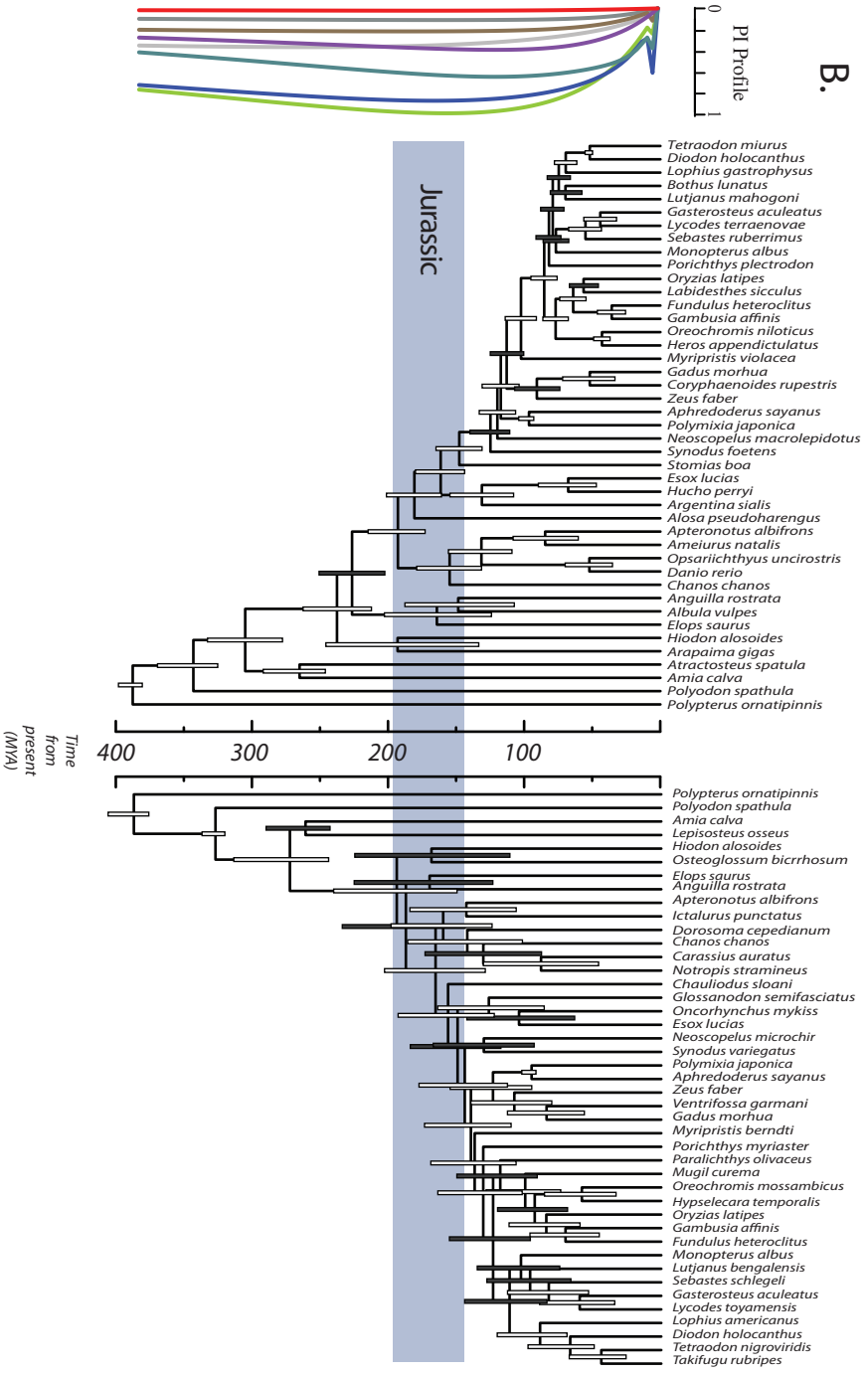

Supplement: Additional file 2: Figure S1. — Comparison of mtDNA and nDNA chronograms for actinopterygians based on A) all data and B) analyses of excluding saturated partitions. Analyses utilized all calibrations except the cichlid calibration based on the interval of paleontological age estimates in Friedman et al. [64]. Bars indicate 95% HPD intervals of age estimates. Light bars indicate posterior probabilities greater than 0.95. Gray bars indicate posterior probabilities below 0.95. Phylogenetic informativeness profiles for both datasets are shown adjacent to the associated chronograms. Colours identify individual partitions. [file s12862-014-0169-0-S2.pdf]

A.

## Nuclear Genes

## Mitochondrial Genes

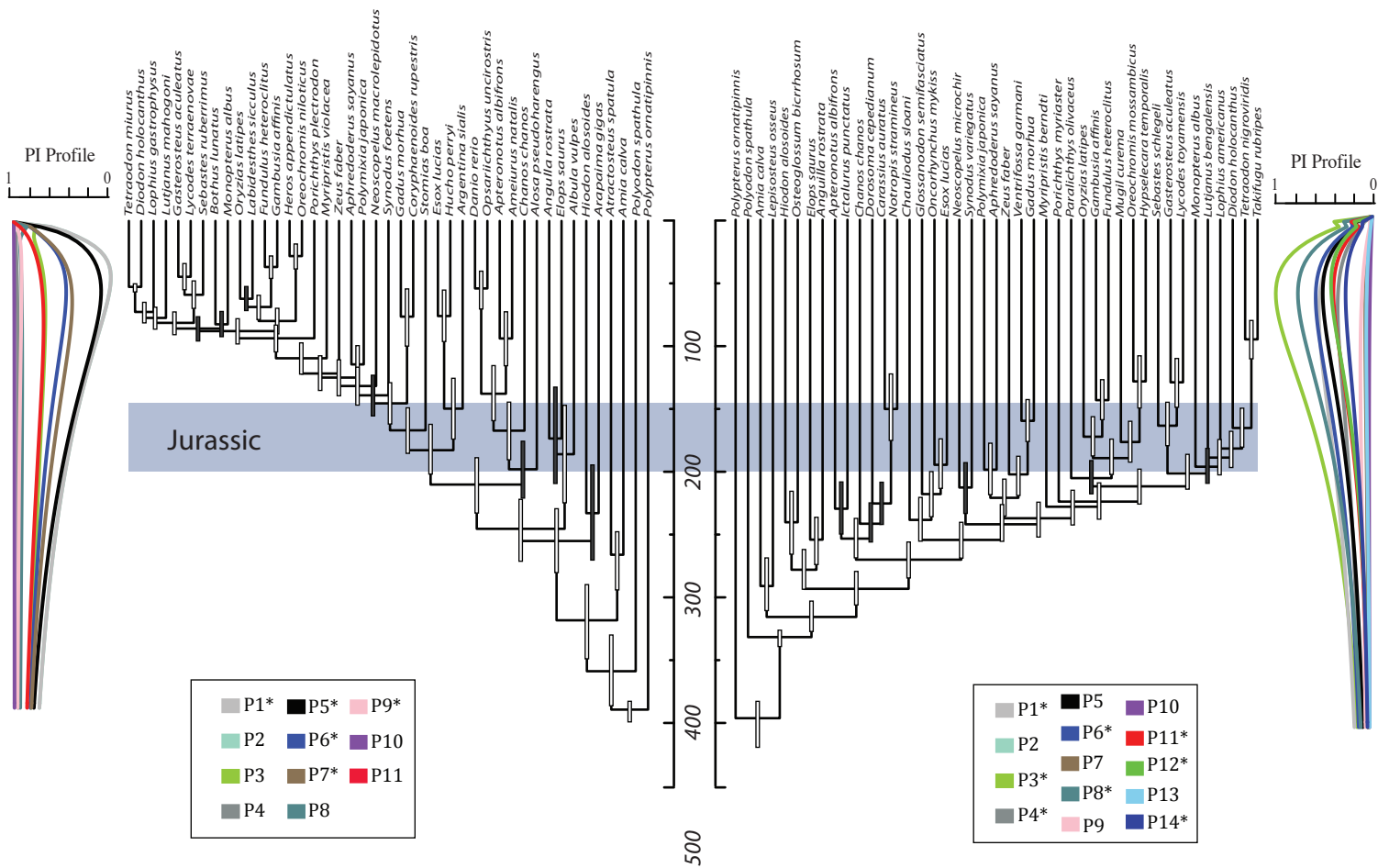

B.

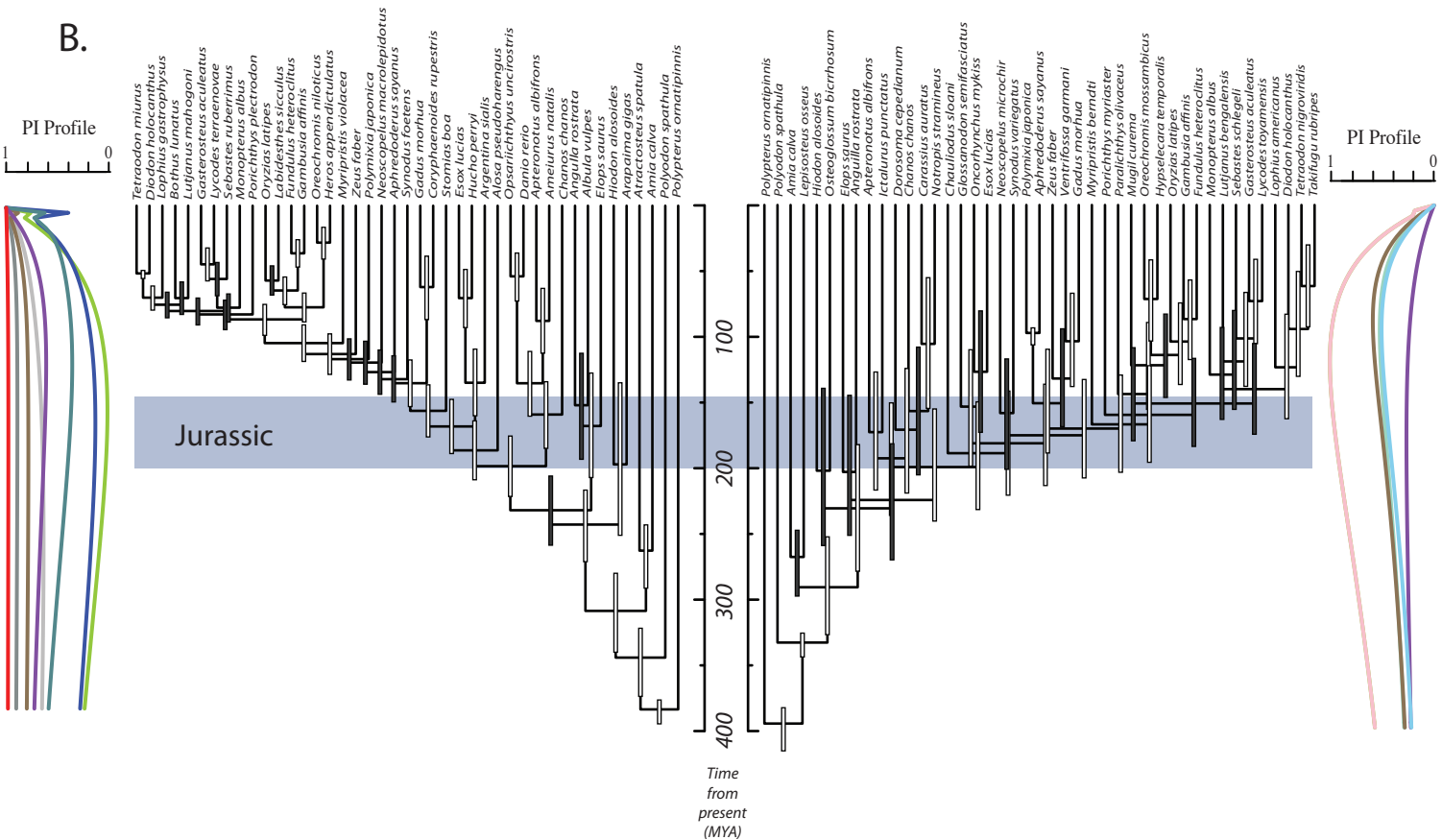

Supplement: Additional file 3: Figure S2. — Comparison of mtDNA and nDNA chronograms for actinopterygians based on A) all data and B) analyses of excluding saturated partitions. Analyses utilized all calibrations except the stem Tetraodontiform and cichlid calibration. Bars indicate 95% HPD intervals of age estimates. Light bars indicate posterior probabilities greater than 0.95. Gray bars indicate posterior probabilities below 0.95. Phylogenetic informativeness profiles for both datasets are shown adjacent to the associated chronograms. Colours identify individual partitions. [file s12862-014-0169-0-S3.pdf]

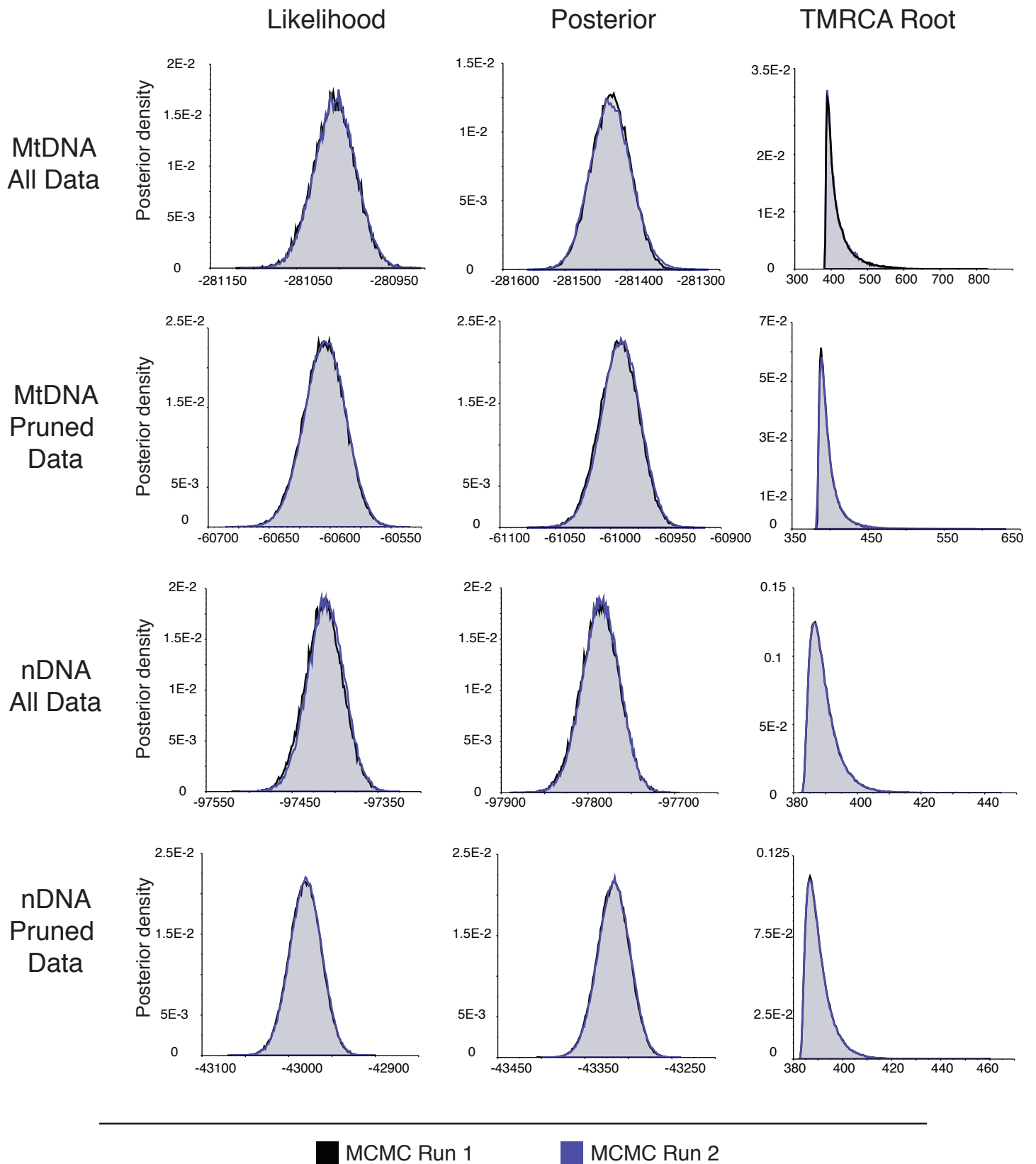

Supplement: Additional file 4: Figure S3. — Visualizations of the Bayesian posterior density between replicate MCMC runs for selected parameters and different DNA datasets. [file s12862-014-0169-0-S4.pdf]
